# Comprehensive comparison between azacytidine and decitabine treatment in an acute myeloid leukemia cell line

**DOI:** 10.1186/s13148-022-01329-0

**Published:** 2022-09-11

**Authors:** Tina Aumer, Constanze B. Gremmelmaier, Leander S. Runtsch, Johannes C. Pforr, G. Nur Yeşiltaç, Stefanie Kaiser, Franziska R. Traube

**Affiliations:** 1 Department of Chemistry, Institute for Chemical Epigenetics, Ludwig-Maximilians-Universität München, Würmtalstr. 201, 81375 Munich, Germany; 2 Faculty of Chemistry, Technical University of Munich, Lichtenbergstr. 4, 85748 Garching, Germany; 3 Institut Für Pharmazeutische Chemie, Goethe-Universität Frankfurt Am Main, Max-von-Laue-Straße 9, 60438 Frankfurt, Germany; 4 Computational Systems Biochemistry Research Group, Max Planck Institute of Biochemistry, Martinsried, Germany

**Keywords:** DNA hypomethylating agents, Epigenetic drugs, Cancer epigenome, 5-aza-cytidine, 5-aza-2’-deoxycytidine

## Abstract

Azacytidine (AzaC) and decitabine (AzadC) are cytosine analogs that covalently trap DNA methyltransferases, which place the important epigenetic mark 5-methyl-2’-deoxycytidine by methylating 2’-deoxycytidine (dC) at the C5 position. AzaC and AzadC are used in the clinic as antimetabolites to treat myelodysplastic syndrome and acute myeloid leukemia and are explored against other types of cancer. Although their principal mechanism of action is known, the downstream effects of AzaC and AzadC treatment are not well understood and the cellular prerequisites that determine sensitivity toward AzaC and AzadC remain elusive. Here, we investigated the effects and phenotype of AzaC and AzadC exposure on the acute myeloid leukemia cell line MOLM-13. We found that while AzaC and AzadC share many effects on the cellular level, including decreased global DNA methylation, increased formation of DNA double-strand breaks, transcriptional downregulation of important oncogenes and similar changes on the proteome level, AzaC failed in contrast to AzadC to induce apoptosis efficiently in MOLM-13. The only cellular marker that correlated with this clear phenotypical outcome was the level of hydroxy-methyl-dC, an additional epigenetic mark that is placed by TET enzymes and repressed in cancer cells. Whereas AzadC increased hmdC substantially in MOLM-13, AzaC treatment did not result in any increase at all. This suggests that hmdC levels in cancer cells should be monitored as a response toward AzaC and AzadC and considered as a biomarker to judge whether AzaC or AzadC treatment leads to cell death in leukemic cells.

**Supplementary Information:**

The online version contains supplementary material available at 10.1186/s13148-022-01329-0.

## Background

5-Azacytidine (azacytidine, AzaC) and 5-aza-2’-deoxycytidine (decitabine, AzadC) are cytosine analogs that belong to the compound class of hypomethylating agents and are applied in the clinic against myelodysplastic syndrome (MDS) and acute myeloid leukemia (AML) [1, 2]. Furthermore, there is ongoing research if and how AzaC and AzadC can contribute to the treatment of other types of cancer [3–5]. AzaC and AzadC have a dual mode of action by addressing epigenetic and DNA damage processes. After uptake, the majority of AzaC is incorporated into RNA, where it inhibits the tRNA (cytosine(38)-C(5))-methyltransferase (*TRDMT1*, DNMT2). In addition, it is metabolized on the diphosphate level to the respective AzadC analogue and is subsequently incorporated into DNA [6, 7]. After genomic incorporation, DNA methyltransferases (DNMTs) are inhibited by creating permanent cross-links between the protein and the 5-aza-cytosine nucleobase. On the epigenetic level, this leads to a global loss of the epigenetic mark 5-methyl-2’-deoxycytidine (mdC) as newly synthesized DNA cannot be methylated anymore [1, 8]. Aberrant mdC patterns are one characteristic of many cancer types since mdC in promoter regions results in gene silencing [9, 10]. The induction of massive DNA demethylation in promoter regions of previously silenced tumor suppressor genes might therefore be a mean of clinically reactivating them [11]. DNA–protein cross-links on the other hand are a severe form of DNA damage that must be repaired via Fanconi anemia-dependent homologous recombination (FA pathway) [12]. Unrepaired cross-links lead to a stalled replication fork that eventually collapses and results in DNA double strand breaks (DSBs) that are highly deleterious for untransformed as well as cancer cells and therefore trigger apoptosis [13, 14]. Although the basic chemistry of AzaC and AzadC is known, many aspects of their mode of action are not well understood. It remains unclear how much the incorporation of AzaC into RNA and thus inhibition of DNMT2 and other m^5^C methyltransferases in general contribute to the efficacy of AzaC [8]. While reports show that in some cancer cell lines AzaC is more efficient than AzadC in inducing cell death, other cancer cell lines show the opposite behavior [6, 15]. Even though the direct effects on the mdC levels have been intensively studied, there is a lack of information on how AzaC and AzadC treatment affects the proteome of AML cells. Moreover, not a single reliable biomarker has been found that can predict whether treatment with AzaC or AzadC is effective [16, 17]. Therefore, to fully exploit the potential of 5-aza-cytosines as epigenetic drugs for cancer therapy, it is of great importance to gain deeper knowledge of the molecular patterns that determine which cellular signaling pathways are altered after treatment with AzaC or AzadC.

## Results

### ***Effect of AzaC and AzadC on mdC, m***^***5***^***C and DNA damage in MOLM-13***

**Fig. 1:**
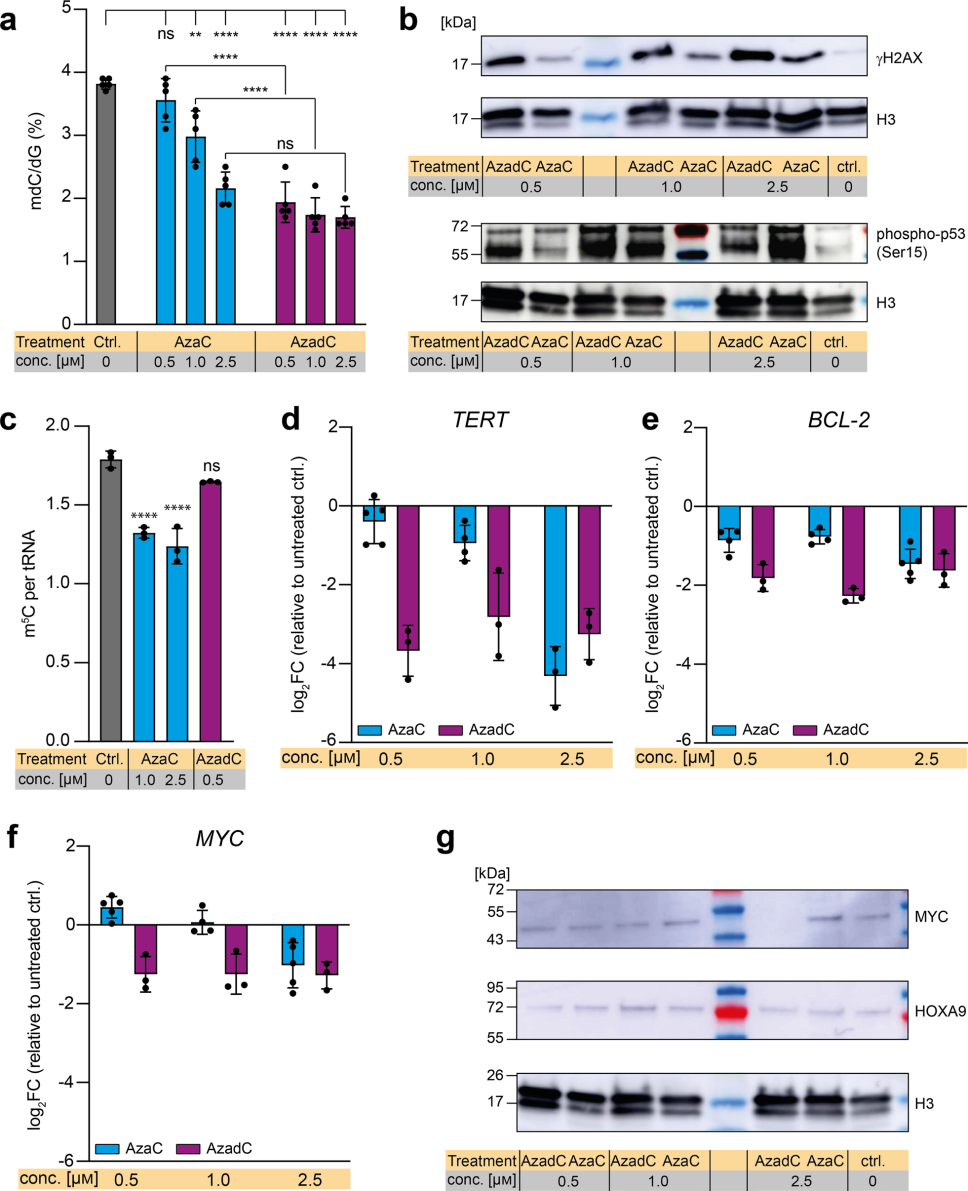
Response of MOLM-13 to AzaC and AzadC treatment on epigenome, DNA damage and gene expression level. **a** UHPLC-QQQ-MS was used to quantify global mdC levels after exposure to 0.5 µM, 1.0 µM or 2.5 µM AzaC or AzadC for 72 h. Untreated cells served as a control. Ordinary one-way ANOVA with Tukey’s multiple comparisons test was performed. **b** Immunoblot analysis of γH2AX and phospho-p53 (Ser15) levels (nuclear fraction) after exposure to 0.5 µM, 1.0 µM or 2.5 µM AzaC or AzadC for 48 h. **c** UHPLC-QQQ-MS was used to measure the m^5^C content per tRNA after exposure to 1.0 µM or 2.5 µM AzaC or 0.5 µM AzadC for 72 h. Untreated cells served as a control. Ordinary one-way ANOVA with Dunnett’s multiple comparisons test was performed. **d–f** RT-qPCR data to quantify gene expression on the transcript level of TERT (**d**), BCL-2 (**e**) and MYC (**f**), after 72 h exposure to 0.5 µM, 1.0 µM or 2.5 µM AzaC or AzadC. The log_2_ fold changes (log_2_FC) of the transcripts in relation to the untreated control are displayed. **g** Immunoblot analysis of MYC and HOXA9 protein levels after exposure to 0.5 µM, 1.0 µM or 2.5 µM AzaC or AzadC for 48 h **a**, **c–f** Each dot represents one independent experiment. Bars show mean, and error bars represent standard deviation. All *p* values were adjusted for multiple comparisons testing. ns *p*_adj_ ≥ 0.05, *p_adj_ < 0.05, **p_adj_ < 0.01, ***p_adj_ < 0.001, ****p_adj_ < 0.0001. **b**, **g** Histone H3 served as a loading ctrl. Untreated cells served as a biological control. **a** Details about the analysis, including exact* p* values, are given in Additional file 10: Table S9. **b** Details about the analysis, including exact *p* values, are given in Additional file 11: Table S10

To compare the effects of AzaC and AzadC treatment, we performed a comprehensive analysis on the cellular phenotype and the underlying molecular changes between AzaC- and AzadC-treated cells. As a model cell line, we chose the AML cell line MOLM-13 because previous studies reported an effective response to AzaC as well as AzadC treatment on DNA methylation level [7, 18], confirming that both compounds are taken up and metabolized correctly. First, we checked by ultra-HPLC triple quadrupole mass spectrometry (UHPLC-QQQ-MS) whether the global mdC level changed upon AzaC and AzadC treatment as expected. In accordance with previously published results [7], mdC levels decreased after exposure to AzaC and AzadC for 72 h (Fig. 1a). For AzaC, the decrease was concentration dependent with 0.5 µM of AzaC being insufficient to reduce mdC levels significantly compared to the control. In contrast, mdC levels dropped substantially after exposure to 0.5 µM, 1.0 µM and 2.5 µM of AzadC, without difference between the concentrations. When comparing the change of mdC levels after exposure to equal amounts of the two compounds, only the highest dose of AzaC (2.5 µM) reduced the mdC levels to a similar extent as AzadC (Fig. 1a). Next, we assessed the level of DNA damage that is introduced by AzaC and AzadC. Therefore, we investigated whether AzaC and AzadC treatment leads to DSB formation in a dose-dependent manner in MOLM-13 by immunoblot analysis against γH2AX (Fig. 1b, Additional file 1: Fig. S1a). H2AX is a histone variant that is placed at DSB sites and subsequently phosphorylated at Ser-139 (γH2AX) to mark the site where the strand breaks occurred and subsequently recruit the repair machinery [19]. Since one γH2AX is placed per DSB, it is a very sensitive and quantitative marker for DSB formation. For AzadC, we observed a substantial increase in γH2AX levels for all three concentrations tested, whereby treatment with 2.5 µM AzadC seemed to result in slightly more DSBs than treatment with 0.5 µM and 1.0 µM. Treatment with AzaC also lead to DSB formation, although to a lesser extent than AzadC treatment and in a strictly concentration-dependent manner. As DNA damage induces phosphorylation and thereby activation of cellular tumor antigen p53, with the initial phosphorylation taking place at serine 15 [20, 21], we also checked the levels of phospho-p53 (Ser15) as response to AzaC and AzadC treatment in MOLM-13 (Fig. 1b, Additional file 1: Fig. S1b). The basal level of phospho-p53 (Ser15) was low (Fig. 1b ctrl.), whereas treatment with AzaC and AzadC both stimulated Ser15 phosphorylation of p53 substantially. With 0.5 µM AzaC, the level of p53 activation was only moderate. Interestingly, however, we obtained the highest level of phospho-p53 (Ser15) with 2.5 µM AzaC among all treatments tested (Fig. 1b). Next, we measured the amount of 5-methyl-cytidine (m^5^C) per tRNA (Fig. 1c). m^5^C is the product of DNMT2, which methylates cytidine 38 in tRNA^Asp^ [22] and NSUN2, which methylates various tRNAs at position 34 and 48–50 [23]. We observed the anticipated reduction of m^5^C in tRNA after exposure to AzaC, but not after AzadC treatment.

### Transcriptional repression of important oncogenes after AzaC and AzadC treatment

After confirming that treatment with either AzaC or AzadC had the expected effect on MOLM-13 on the epigenetic and DNA damage level, we wanted to further characterize how treatment influenced the phenotype of MOLM-13. Therefore, we investigated how the transcript levels of three important (proto-)oncogenes, namely the telomerase reverse transcriptase TERT (Fig. 1d, Additional file 1: Fig. S1c), the apoptosis inhibitor BCL-2 (Fig. 1e, Additional file 1: Figure S1d) and the transcription factor MYC (Fig. 1f, Additional file 1: Fig. S1e), all three being main drivers of tumorigenesis and progression [24], changed upon treatment with AzaC and AzadC. *TERT* and *BCL-2* expression were substantially downregulated after AzaC treatment at 2.5 µM (log_2_FC *TERT* -3.3, log_2_FC *BCL-2* -1.5) and we observed that AzaC was able to repress *MYC* expression only at the highest concentration (log_2_FC -1.5). In contrast, AzadC treatment was effective in downregulating *TERT*, *BCL-2* and *MYC* expression at all three concentrations. Importantly, at 2.5 µM, AzaC and AzadC showed a comparable reducing effect on the transcript level of all three genes. These results were in line with the UHPLC-QQQ-MS mdC and the γH2AX western blot data that revealed a similar effect of 2.5 µM AzaC and AzadC treatment. On the protein level, however, only 2.5 µM of AzadC were able to reduce MYC expression levels with MYC not being detectable at all in the immunoblot analysis (Fig. 1g, Additional file 1: Fig. S1f). This observation indicated a compensating mechanism in the MOLM-13 to keep the MYC protein level high despite reduced *MYC* transcription at lower concentrations of AzadC and at 2.5 µM AzaC. In contrast, the protein expression levels of the transcription factor HOXA9, which is important for leukemic transformation and consequently often upregulated in AML [25], did respond alike after AzaC and AzadC treatment and were even increased at treatment with 1.0 µM of either compound (Fig. 1g, Additional file 1: Fig. S1g).

### Induction of proliferation stop and apoptosis with low doses of AzadC, but not AzaC in MOLM-13

**Fig. 2:**
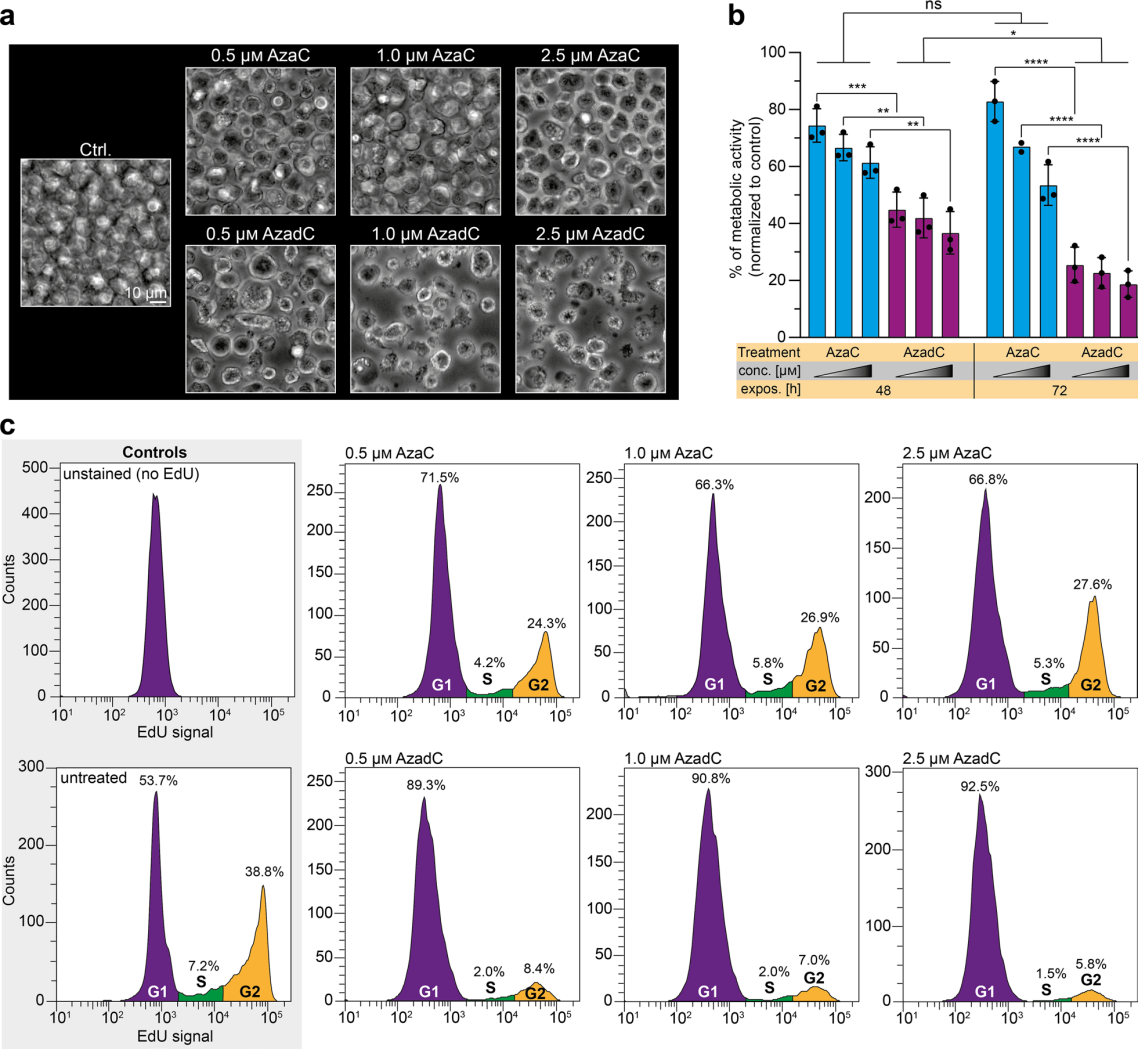
Monitoring of cellular fitness and proliferation of MOLM-13 after treatment with AzaC or AzadC.** a** Brightfield microscopy images of MOLM-13 treated for 72 h with different concentrations of either AzaC or AzadC. Untreated cells served as a control. **b** MTT assay results to measure the metabolic activity after 48 h or 72 h exposure to 0.5 µM, 1.0 µM and 2.5 µM (increasing concentrations are indicated by the triangle) AzaC or AzadC. The metabolic activity of treated cells was individually normalized to the metabolic activity of the untreated control for every independent experiment. Each dot represents one independent experiment. Ordinary two-way ANOVA combined with Tukey’s multiple comparisons test was performed. All *p* values were adjusted for multiple comparisons testing. ns *p*_adj_ ≥ 0.05, *p_adj_ < 0.05, **p_adj_ < 0.01, ***p_adj_ < 0.001, ****p_adj_ < 0.0001. **c** Flow cytometric analysis of proliferation using EdU after 48 h of treatment with 0.5 µM, 1.0 µM and 2.5 µM of AzaC or AzadC. On the x-axis the signal intensity of the EdU, which has been conjugated to Alexa488 by click chemistry, is displayed for each detected cell. The count (y-axis) displays how many cells with a certain EdU signal intensity have been detected. Cells that had an EdU signal intensity > 1.5 × 10^4^ were considered to have completed S-phase and reached G2 phase (orange area), EdU intensity > 2 × 10^3^, but < 1.5 × 10^4^ was considered as S-phase (green) and EdU signal < 2 × 10^3^ represents cells that had not entered S-phase within the two hours of EdU exposure (violet area). **b** Details about the analysis, including exact p values, are given in Additional file 12: Table S11

Based on these results, a similar effect of AzaC treatment, at least at the highest concentration of 2.5 µM AzaC, and AzadC treatment at all three concentrations on the MOLM-13 cells was expected. Therefore, we monitored the phenotype of MOLM-13 after AzaC and AzadC treatment by brightfield microscopy (Fig. 2a). As an AML cell line, MOLM-13 are suspension cells and show a clearly defined round shape with a diameter of 10–15 µm. Surprisingly, the cells mostly contained their defined round shape and cellular diameter after AzaC treatment at all three concentrations, but seemed to have a moderate increase in granularity and to proliferate slower compared to the untreated cells. In complete contrast, AzadC treatment at all three concentrations had a severe effect on MOLM-13 morphology and growth. Only a tiny fraction retained the described round shape and anticipated diameter, but most cells instead had dramatically shrunk, indicating advanced apoptosis. A few cells showed enlarged cell size, which is a sign of beginning necrosis (Fig. 2a). When we tested the metabolic activity of MOLM-13 in an MTT assay, which measures metabolic activity to estimate cell viability, proliferation and overall fitness, after treatment with AzaC or AzadC for either 24 h, 48 h or 72 h in a concentration-dependent manner, we also noted substantial differences between the two treatments (Fig. 2b). While the reduction of metabolic activity was only moderate for all tested treatments after 24 h (Additional file 1: Fig. S2a), the metabolic activity had decreased significantly in a concentration-dependent manner for all treatments by 48 h. Whereas there was no significant difference between AzaC- and AzadC-treated cells at the earlier time point, after 48 h, AzadC treatment was significantly more effective than AzaC treatment. The average reduction of metabolic activity was between 30 and 40% for AzaC and between 60 and 70% for AzadC. When exposed for 72 h, no significant additional decrease in metabolic activity in the AzaC-treated cells could be observed. However, cells that were treated with AzadC showed an even more drastic drop of metabolic activity of about 80% compared to the control (Fig. 2b). In summary, the MTT assay revealed that AzaC treatment had an initial negative impact on the fitness of the MOLM-13, but metabolic activity could not be reduced further than 50% and only when the highest concentration of 2.5 µM was applied. In contrast, AzadC treatment resulted in a reduction of metabolic activity by 80% for all concentrations tested. Next, we investigated how AzaC and AzadC affected the proliferation rate of MOLM-13 after 48 h of treatment using 5-ethynyl-2’-deoxyuridine (EdU), which can be conjugated to a fluorophore via click chemistry after feeding. EdU was applied for two hours and the amount of cells that incorporated EdU and reached G2 phase was afterward quantified by flow cytometry. Intriguingly, AzaC treatment slowed down proliferation only to a small extent compared to the untreated control, independent from the applied concentration, whereas MOLM-13 that were treated with AzadC hardly proliferated anymore even at the lowest concentration of 0.5 µM (Fig. 2c).

**Fig. 3:**
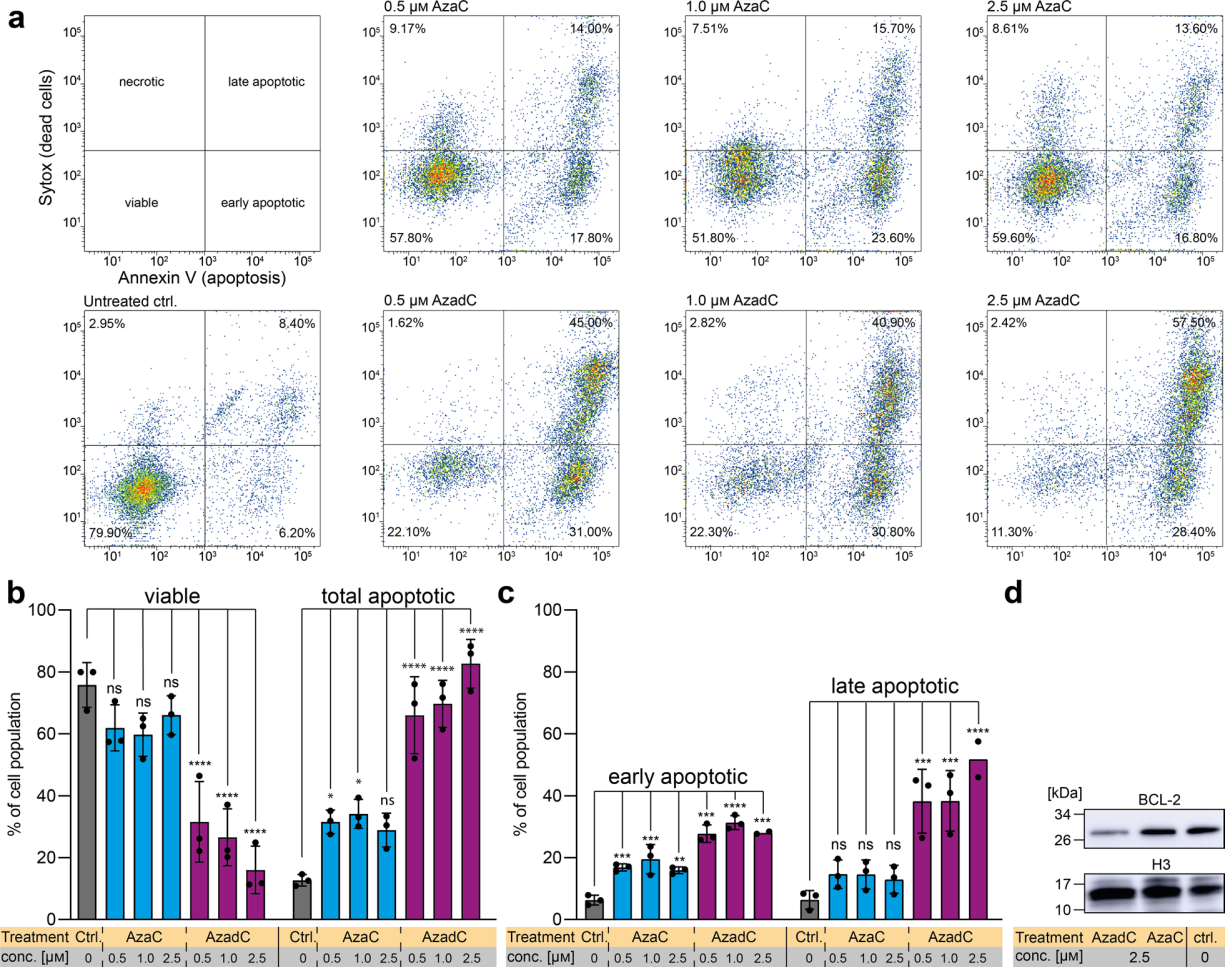
Monitoring of cell death event of MOLM-13 after treatment with AzaC or AzadC. **a** Flow cytometric analysis of cell death after 72 h treatment with either AzaC or AzadC in different concentrations. Cells that were Annexin V low and Sytox™ low cells were considered viable, cells only high in Sytox as necrotic, cells only high in Annexin V as early apoptotic and cells high in both as late apoptotic. **b** Quantification of three independent flow cytometric cell death analyses performed as depicted in (**a**) was applied, combining all Annexin V high cells as total apoptotic. **c** Differential analysis of early and late apoptotic events of **b**. **d** Immunoblot analysis of BCL-2 protein levels upon AzaC and AzadC treatment (2.5 µM each for 72 h) as compared to untreated control. Histone H3 served as a loading control. **b**, **c** Each dot represents one independent experiment. Bars show mean, and error bars represent standard deviation. Ordinary one-way ANOVA combined with Dunnett’s multiple comparisons test was performed within each observed group (viable and apoptotic in **b**, early and late apoptotic in **c**). All p values were adjusted for multiple comparisons testing. ns *p*_adj_ ≥ 0.05, *p_adj_ < 0.05, **p_adj_ < 0.01, ***p_adj_ < 0.001, ****p_adj_ < 0.0001. **b** Details about the analysis, including exact p values, are given in Additional file 13: Table S12.** c**: Details about the analysis, including exact p values, are given in Additional file 14: Table S13

In a next step, we quantified and characterized the type of AzaC or AzadC caused cell death after 72 h of treatment by flow cytometry using a combination of the apoptosis marker Annexin V and a live/dead cell stain (Sytox™) (Fig. 3a–c). Annexin^−^/Sytox^−^ cells were considered as viable, Annexin^−^/Sytox^+^ cells as necrotic, Annexin^+^/Sytox^−^ cells as early and Annexin^+^/Sytox^+^ cells as late apoptotic (Fig. 3a). As expected, the majority of the untreated cells were viable with minor populations showing early and late apoptosis and very few undergoing necrosis (Fig. 3a, b). In line with the brightfield microscopy images, AzaC-treated cells showed no significant decrease in viability in the flow cytometric analysis. Treatment with the highest concentration of 2.5 µM AzaC did not result in a statistically significant increase in overall apoptotic events compared to the control (Fig. 3b) with cells in the late apoptotic state being absent after 72 h exposure to AzaC at any of the concentrations tested (Fig. 3c). The anticipated increase in granularity from the brightfield microscopy images was, however, not confirmed (Additional file 1: Fig. S2b). In contrast, AzadC treatment at all three concentrations resulted in a significant accumulation of cells in an early and late apoptotic, but not in a necrotic state (Fig. 3a–c). Remarkably, the majority of cells was already in a late apoptotic state upon AzadC treatment at only 0.5 µM (Fig. 3a, c). One of the master regulators of apoptosis is BCL-2, which prevents cells from entering apoptosis and is therefore highly expressed in most cancer cells [24]. On transcript level, *BCL-2* was downregulated to the same extent upon application of 2.5 µM AzaC or AzadC, respectively (Fig. 1e), suggesting that apoptosis can be induced after both treatments. However, when analyzing the amount of protein by immunoblot, we observed that 2.5 µM of AzaC failed to downregulate BCL-2 on the protein level in contrast to 2.5 µM AzadC (Fig. 3d, Additional file 1: Fig. S2c), indicating that information about the changes on the protein level is essential to judge the effectivity of the treatment. In summary, our data about the cell death events on single-cell level, obtained by flow cytometry, showed that data from MTT or similar assays, which do not determine cell death directly, are not suitable as a stand-alone method to determine viability of cancer cells because the metabolic activity can be influenced by several parameters. In our case, the effects of AzaC on MOLM-13 viability would have been drastically overestimated by the MTT assay.

### Proteome changes after AzaC and AzadC treatment reveal similar, but not identical changes in expression patterns

**Fig. 4:**
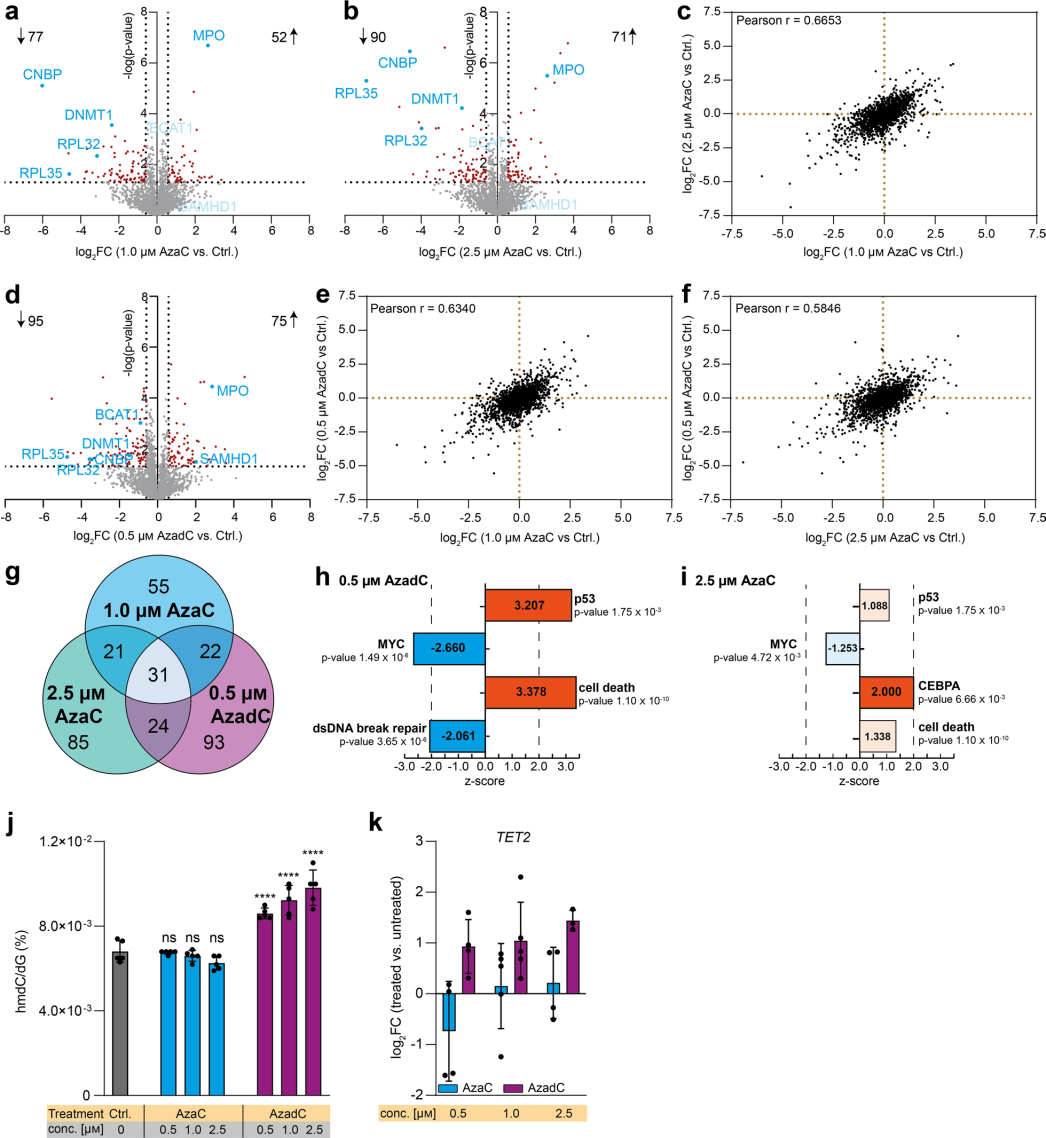
Full-proteome analysis of MOLM-13 and TET activity after 72 h AzaC or AzadC treatment. **a, b** Volcano plot of differentially expressed proteins of **a** 1.0 µM AzaC and **b** 2.5 µM AzaC-treated cells versus untreated control. **c** Correlation plot where the log_2_FC after treatment with 1.0 µM AzaC and 2.5 µM AzaC is displayed for each protein without considering the p value for the enrichment or depletion. **d** Volcano plot of differentially expressed proteins of 0.5 µM AzadC-treated cells versus untreated control. **e** Correlation plot where the log_2_FC after treatment with 1.0 µM AzaC and 0.5 µM AzadC is displayed for each protein without considering the *p* value for the enrichment or depletion. **f** Correlation plot where the log_2_FC after treatment with 2.5 µM AzaC and 0.5 µM AzadC is displayed for each protein without considering the p value for the enrichment or depletion. **g** Venn diagram of the differentially expressed proteins after 1.0 µM AzaC, 2.5 µM AzaC and 0.5 µM AzadC treatment. Commonly differentially expressed proteins are indicated by the overlap between two or more treatments. **h** Results of the Ingenuity Pathway Analysis (IPA) of the proteome changes of AzadC (0.5 µM)-treated MOLM-13 compared to the untreated control. **i** Results of the IPA of the proteome changes of AzaC (2.5 µM)-treated MOLM-13 compared to the untreated control. **j** UHPLC-QQQ-MS was used to quantify global hmdC levels after exposure to 0.5 µM, 1.0 µM or 2.5 µM AzaC or AzadC for 72 h. Untreated cells served as a control. Ordinary one-way ANOVA with Tukey’s multiple comparisons test was performed. ns *p*_adj_ ≥ 0.05, ****p_adj_ < 0.0001. **k** RT-qPCR data to quantify gene expression on the transcript level of TET2 after 72 h exposure to 0.5 µM, 1.0 µM or 2.5 µM AzaC or AzadC. The log_2_ fold changes (log_2_FC) of the transcripts in relation to the untreated control are displayed. **a**, **b**, **d** For each treatment (1.0 µM AzaC, 2.5 µM AzaC, 0.5 µM AzadC, untreated ctrl), four biologically independent experiments were performed and measured. Proteins were considered as differentially expressed when the criteria log_2_FC >|0.58496| (fold change >|1.5|) and -log(*p* value) > 1.301 (*p* value < 0.05) were both fulfilled. Left side of volcano plot: proteins depleted after Aza treatment, right side of volcano plot: proteins enriched after Aza treatment. **c**, **e**, **f** log_2_FC for each detected protein of (**a**, **b**, **d**), regardless of the *p* value, in comparison with each other. Pearson correlation coefficient was calculated. **i, j** *p* value indicates *p* value of overlap for the Fisher’s Exact Test. The number in the bars displays the z-score, a z-score > 0 indicates activation, z-score < 0 indicates inhibition. Z-score ≥|2| was considered as significantly activated or inhibited, respectively. P53, MYC and CEBPA are part of the “Upstream Regulator Analysis.” Cell death and dsDNA break repair are part of the “Disease and function” analysis. Detailed results are listed in Additional file 6 Table S5. **j**, **k** Bar shows mean, and error bars show standard deviation. Each dot represents one biologically independent experiment

Since neither the mdC levels, the m^5^C levels, the amount of DNA damage nor the transcript levels of three important oncogenes turned out to be good AzaC or AzadC response markers in MOLM-13 regarding cell death, we aimed to identify other possible cellular markers with better predictive value. To this end, we performed a comprehensive analysis of the proteome changes that were induced by AzaC or AzadC treatment in MOLM-13. We analyzed the proteome changes after 72 h of treatment with drug concentrations that caused a low to moderate induction of cell death, namely 1.0 µM or 2.5 µM AzaC or 0.5 µM AzadC. In total, 129 proteins were differentially expressed compared to the untreated control after exposure to 1.0 µM (Fig. 4a, Additional file 2: Table S1) and 161 proteins after exposure to 2.5 µM AzaC (Fig. 4b, Additional file 3: Table S2). Regarding the fold change of all detected proteins, there was a significant positive correlation (Pearson r = 0.6653, Fig. 4c), indicating that AzaC treatment with the two different concentrations had a very similar, but not the same effect on the cells. After 0.5 µM AzadC treatment, 170 proteins were differentially expressed (Fig. 4d, Additional file 4: Table S3), among them 95 proteins being downregulated and 75 proteins being upregulated, which was very similar in terms of numbers to 2.5 µM AzaC treatment, where 90 proteins were downregulated and 71 proteins upregulated. Surprisingly, the correlation between the fold changes of the detected proteins was very similar between 1.0 µM AzaC and 0.5 µM AzadC treatment (Pearson *r* = 0.6340, Fig. 4e) and 2.5 µM AzaC and 0.5 µM AzadC treatment (Pearson *r* = 0.5846, Fig. 4f) compared to the correlation between both AzaC treatments (Pearson *r* = 0.6653, Fig. 4c). Hierarchical clustering revealed that while all four independent replicates of the untreated control clustered together, there was no such clear clustering of the AzaC- and AzadC-treated cells (Additional file 1: Fig. S3). A closer analysis on the individual differentially expressed proteins revealed that all three treatments (1.0 µM AzaC, 2.5 µM AzaC and 0.5 µM AzadC) resulted in common differential expression of 31 proteins compared to the untreated control (Fig. 4g, Table 1) and the direction of differential expression (up- or downregulated) was the same. As expected, DNMT1 was significantly downregulated after all three treatments (Fig. 4a, b, d, Table 1). Myeloperoxidase (MPO), a marker of myeloid lineage commitment, on the other hand was highly upregulated after all three treatments. This is in accordance with literature as the promoter region of MPO is known to be demethylated by DNMT-inhibitors like decitabine, which increases transcription of the *MPO* gene [26]. One of the proteins that showed the highest fold change in all three data sets was the cellular nucleic acid binding protein (CNBP) (Fig. 4a, b, d, Table 1). CNBP is a DNA- and RNA-binding protein that was shown to unfold G-quadruplex structures in promoters of several important oncogenes, thereby promoting their transcription translation on a global scale, among them MYC [27, 28]. Furthermore, two ribosomal proteins, RPL32 and RPL35, were substantially downregulated after all three treatments (Fig. 4a, b, d, Table 1). We next computed the significance of the overlap of commonly differentially expressed proteins using DynaVenn [29]. The overlap was significant for all treatments and also in a pairwise comparison (*p* value < 0.0001). For commonly differentially expressed proteins, the direction of the regulation was always the same. These results indicated that AzaC and AzadC treatment did not result in fundamentally different proteome changes. When analyzing the 93 proteins that were uniquely differentially expressed after 0.5 µM AzadC treatment, there was no enrichment for a specific pathway being altered by AzadC treatment when directly compared to AzaC treatment. Rather, AzadC treatment changed the expression of several proteins that are involved in very different cellular pathways, including chromatin organization and heterochromatin formation, transcription and translation, proteasome function and metabolism (Additional file 4: Table S3). Interestingly, the deoxynucleoside triphosphate triphosphohydrolase SAMHD1 was among those proteins that were significantly higher expressed after 0.5 µM AzadC treatment only (Fig. 4a, b, d). SAMHD1 is a triphosphate hydrolase that specifically deactivates the AzadC triphosphate, but not the AzaC triphosphate and is therefore one of the reasons why some AML subtypes do not respond to AzadC [30]. However, AzadC treatment resulted in rapid cell death of MOLM-13 despite a fourfold (log_2_FC ~ 2) upregulation of SAMHD1, which suggests that the dosage of AzadC must initially be high enough to induce rapid cell death in MOLM-13 before they can efficiently develop resistance mechanisms. In a next step, we performed a comprehensive pathway analysis based on the proteome changes in the 0.5 µM AzadC and the 2.5 µM AzaC treated MOLM-13 compared to the untreated control, respectively, using the Ingenuity Pathway Analysis tool (IPA) [31]. For the input data (Additional file 5: Table S4), we set a *p* value cutoff of 0.05 for the protein expression changes and treated all other detected proteins as background. Additionally, we distinguished between proteins that were up- or downregulated but detectable in all samples from those that were uniquely detectable in either the control or the treated samples. The reason for this distinction was that the anticipated impact on the cellular phenotype of proteins whose expression was exclusively activated or repressed following AzadC or AzaC treatment was different from the impact of proteins that showed changes in the expression levels but were present among all samples. For IPA, an absolute z-score equal to or greater than two and a *p* value smaller than 0.05 for the Fisher’s exact test was considered as significant. In accordance with the observed activation of p53 by phosphorylation (Fig. 1b) and as expected as a cellular response to the massive DNA damage, IPA predicted activation of p53 signaling after AzadC treatment (Fig. 4h). In contrast, MYC signaling was predicted to be inhibited (Fig. 4h), which is also indicated by the observed proliferation stop after AzadC treatment (Fig. 2c) and p53 activation, which negatively affects MYC signaling. Interestingly, double-stranded DNA break repair was predicted to be significantly decreased based on the proteomics data, although DSBs were highly increased (Fig. 1b), which indicated that the DNA damage induced by AzadC was too severe to be repaired. As a consequence and in line with the flow cytometric analysis of cell death events (Fig. 3a), cell death of MOLM-13 was expected to be strongly increased based on the proteome changes (Fig. 4h). After AzaC treatment, in contrast, cell death was predicted to be increased, but the level of activation failed to reach the significance threshold for the z-score (Fig. 4i). Moreover, neither p53 nor MYC signaling was significantly affected by AzaC. In contrast, the CEBPA pathway was predicted to be significantly activated (Fig. 4i). CEBPA is the CCAAT/enhancer-binding protein-alpha and is a pivotal transcription factor during hematopoiesis that promotes early myeloid differentiation [32]. CEBPA signaling did not seem to be impacted after AzadC treatment, indicating that there were some cellular pathways which were exclusively affected by either AzaC or AzadC. Nevertheless, in summary, IPA confirmed that AzadC and AzaC treatment had a similar impact on many cellular pathways on the proteome level. However, exposure to AzaC did often not result in a significant activation or inhibition of a certain pathway, including p53, MYC signaling or induction of cell death, whereas exposure to AzadC did, which can explain the substantial phenotypic differences regarding proliferation and cell viability of MOLM-13 after AzadC or AzaC treatment.

**Table 1:** Common differentially expressed proteins in MOLM-13 compared to the untreated control after exposure to either 0.5 µM AzadC or 1.0 µM AzaC or 2.5 µM AzaC for 72 h

Protein	Uniprot ID	log_2_ FC		
		1.0 µM AzaC	2.5 µM AzaC	0.5 µM AzadC
CNBP (CCHC-type zinc finger nucleic acid binding protein)	P62633	− 6.02	− 4.59	− 3.45
ATP5D (ATP synthase subunit delta, mitochondrial)	P30049	− 4.64	− 5.14	− 3.62
RPL35 (60S ribosomal protein L35)	P42766	− 4.60	− 5.14	− 3.62
RPL32 (60S ribosomal protein L32)	P62910	− 3.68	− 3.98	− 3.57
PSMB6 (Proteasome subunit beta type-6)	P28072	− 3.38	− 3.69	− 3.32
WTAP (Pre-mRNA-splicing regulator WTAP)	Q15007	− 2.39	− 2.76	− 2.15
ATG3 (Ubiquitin-like-conjugating enzyme ATG3)	Q9NT62	− 2.23	− 1.93	− 1.61
DNMT1 (DNA (cytosine-5) methyltransferase 1)	P26358	− 2.19	− 1.87	− 1.49
NOL7 (Nuclear protein 7)	Q9UMY1	− 2.12	− 2.33	− 2.86
PLAUR (Urokinase plasminogen activator surface receptor)	Q03405	− 1.97	− 2.18	− 2.33
RRP1B (Ribosomal RNA processing protein 1 homolog B)	Q14684	− 1.94	− 2.00	− 2.66
IRF8 (Interferon regulatory factor 8)	Q02556	− 1.68	− 1.13	− 1.43
MRPL13 (39S ribosomal protein L13, mitochondrial)	Q9BYD1	− 1.53	− 1.24	− 1.37
PTER (Phosphotriesterase-related protein)	Q96BW5	− 1.40	− 2.00	− 2.35
HBS1L (HBS1-like protein)	Q9Y450	− 1.27	− 1.51	− 1.14
PRRC2C (Protein PRRC2C)	Q9Y520	− 1.22	− 0.75	− 1.22
GPT2 (Alanine aminotransferase 2)	Q8TD30	− 1.12	− 0.99	− 1.09
TNKS1BP1 (182 kDa tankyrase-1-binding protein)	Q9C0C2	− 0.98	− 1.54	− 1.59
SATB1 (DNA-binding protein SATB1)	Q01826	− 0.72	− 0.61	− 0.92
DLD (Dihydrolipoyl dehydrogenase, mitochondrial)	P09622	− 0.65	− 0.63	− 1.48
CYB5R3 (NADH cytochrome b5 reductase 3)	P00387	0.62	0.67	0.70
NUBP2 (Cytosolic Fe-S cluster assembly factor NUBP2)	Q9Y5Y2	1.10	1.33	1.66
PPP6R2 (Serine/threonine-protein phosphatase 6 regulatory subunit 2)	O75170	1.13	1.29	1.31
RSF1 (Remodeling and spacing factor 1)	Q96T23	1.24	1.04	0.91
TKTL1 (Transketolase-like protein 1)	PS1854	1.89	3.00	1.50
PEA15 (Astrocytic phosphoprotein PEA-15)	Q15121	1.94	2.01	0.94
TEX10 (Testis-expressed protein 10)	Q9NXF1	2.01	2.27	1.84
ICT1 (Peptidyl-tRNA hydrolase ICT1, mitochondrial)	Q14197	2.09	1.72	1.38
CFD (Complement factor D)	P00746	2.22	2.21	3.05
MPO (Myeloperoxidase)	P11247	2.68	2.62	2.86
GATD3 (Gln amidotransferase-like class 1 domain-cont. protein 3)	P0DPI2	3.38	3.70	4.58

For all proteins listed, the log(p value) was > 1.30 in all three data sets

In summary, the proteome data showed that AzaC and AzadC treatment had a similar, but overall lower impact on MOLM-13 as indicated by the Pearson correlation coefficients, the overlapping differentially expressed proteins and IPA, but nevertheless AzaC treatment failed to induce apoptosis effectively at the applied concentrations (Fig. 3a–c). This suggests that the accumulative impact of AzadC on various cellular levels was important for cellular fate and not targeting of few defined pathways that were specifically altered by AzadC but not AzaC in MOLM-13.

### hmdC as a possible marker for response to AzaC or AzadC treatment

In the genome, mdC can be further modified by ten–eleven translocation enzymes (TET enzymes), which are α-ketoglutarate (αKG)-dependent dioxygenases that oxidize mdC to 5-hydroxy-methyl-2’-deoxycytidine (hmdC) and further on to 5-formyl-dC and 5-carboxy-dC [33, 34]. It has been shown that cancer cells often have low levels of hmdC and that impaired TET activity, either by reduced TET expression or TET inhibition, promotes leukemogenesis, whereas restored TET activity initiates proliferation stop and differentiation of leukemic cells [35–39]. Therefore, we checked by UHPLC-QQQ-MS how 72 h of AzaC or AzadC treatment changed the global hmdC levels in MOLM-13 and observed that AzaC was not able to increase the hmdC levels at any concentration tested (Fig. 4h). In contrast, AzadC treatment had a concentration-dependent effect. Treatment with 0.5 µM AzadC resulted in a 25% increase, treatment with 1.0 µM AzadC resulted in a 35% increase and treatment with 2.5 µM AzadC resulted in a 45% increase in global hmdC. In contrast to the mdC levels, the hmdC levels therefore correlated with treatment outcome. We further quantified *TET2* transcription levels and observed that AzaC treatment did not lead to increased transcription, but AzadC did (Fig. 4i). Additionally, AzadC, but not AzaC treatment resulted in a significant downregulation of branched-chain aminotransferase 1 (BCAT1) protein (Fig. 4a, b, d). Overexpression of BCAT1 was shown to restrict the αKG pool in hematopoietic cells thereby reducing TET activity and promoting leukemic stem cell formation and maintenance [38]. The increased hmdC levels after AzadC treatment could therefore be a consequence of higher TET expression and higher TET activity.

### Effects of AzaC and AzadC on HL-60

**Fig. 5:**
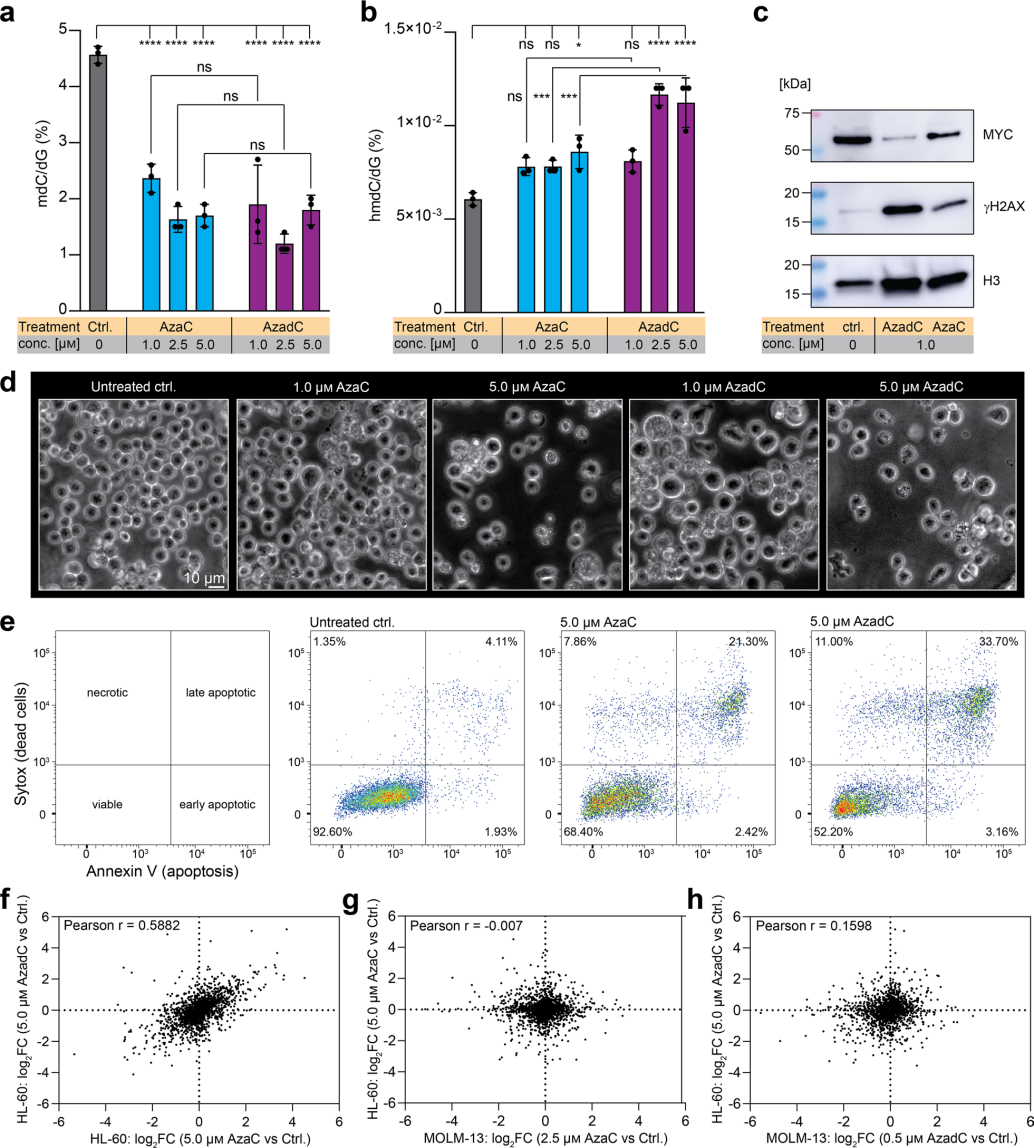
Effects of AzaC and AzadC on the acute promyelocytic leukemia cell line HL-60.** a**,** b** UHPLC-QQQ-MS was used to quantify global mdC (**a**) and hmdC (**b**) levels after exposure to 1.0 µM, 2.5 µM or 5.0 µM AzaC or AzadC for 72 h. Untreated cells served as a control. Ordinary one-way ANOVA with Tukey’s multiple comparisons test was performed. Each dot represents one independent experiment. Bars show mean, and error bars represent standard deviation. All *p* values were adjusted for multiple comparisons testing. ns *p*_adj_ ≥ 0.05, *p_adj_ < 0.05, **p_adj_ < 0.01, ***p_adj_ < 0.001, ****p_adj_ < 0.0001. **c** Immunoblot analysis of γH2AX and MYC (nuclear fraction) after exposure to 1.0 µM AzaC or AzadC for 72 h. **d** Brightfield microscopy images of HL-60 treated for 72 h with different concentrations of either AzaC or AzadC. Untreated cells served as a control. **e** Flow cytometric analysis of cell death after 72 h treatment with either 5.0 µM of AzaC or AzadC. Cells that were Annexin V low and Sytox™ low cells were considered viable, cells only high in Sytox as necrotic, cells only high in Annexin V as early apoptotic and cells high in both as late apoptotic. **f** Correlation plot where the log_2_FC after treatment with 5.0 µM AzaC and 5.0 µM AzadC is displayed for each protein without considering the *p* value for the enrichment or depletion. **g** Correlation plot where the log_2_FC after treatment with 2.5 µM AzaC in MOLM-13 and 5.0 µM AzaC in HL-60 is displayed for each protein without considering the *p* value for the enrichment or depletion. **h** Correlation plot where the log_2_FC after treatment with 0.5 µM AzadC in MOLM-13 and 5.0 µM AzadC in HL-60 is displayed for each protein without considering the *p* value for the enrichment or depletion. **a** Details about the analysis, including exact *p* values, are given in Additional file 15: Table S14. **b** Details about the analysis, including exact *p* values, are given in Additional file 16: Table S15

To extend the analysis of the effects of AzadC and AzaC and compare the results to the ones obtained in MOLM-13, we tested AzadC and AzaC treatment on the acute promyelocytic leukemia cell line HL-60 [40]. For the HL-60, we had to moderately increase the AzaC and AzadC concentration because 0.5 µM was too low for either compound to induce substantial morphological and proliferation changes (Additional file 1: Fig. S4a). First, we quantified the mdC (Fig. 5a) and hmdC (Fig. 5b) levels by UHPLC-QQQ-MS 72 h after treatment with 1.0 µM, 2.5 µM and 5.0 µM of AzaC or AzadC. Both, AzaC and AzadC treatment resulted in a dramatic decrease in mdC with no significant differences between the two compounds (Fig. 5a). In contrast to MOLM-13, however, AzaC treatment resulted in the HL-60 in a significant increase in hmdC at the highest concentration applied (Fig. 5b). Exposure to 2.5 µM or 5.0 µM AzadC resulted in significantly higher hmdC levels compared to the untreated control and to AzaC-treated cells at the same concentration. Interestingly, 1.0 µM of AzadC failed to increase hmdC significantly when compared among all treatments tested (Fig. 5b), whereas in MOLM-13, 0.5 µM of AzadC were enough to induce TET activity (Fig. 4h). Nevertheless, a concentration of only 1.0 µM of AzaC or AzadC was sufficient to substantially induce DSBs and reduce MYC on the protein level as indicated by the immunoblot analysis (Fig. 5c, Fig. S4b). Regarding survival and proliferation, we observed a concentration-dependent decrease after 72 h of AzaC and AzadC treatment (Fig. 5d) with 1.0 µM of AzaC showing no increase in cell death and 1.0 µM of AzadC resulting only in a moderate increase in dead cells. This result was in sharp contrast to the effects of AzadC in MOLM-13, where 0.5 µM of AzadC already induced massive cell death and a complete stop of proliferation (Figs. 2a,c and 3a–c). At 5.0 µM concentration, AzaC and AzadC both induced cell death and flow cytometric analysis confirmed that not only AzadC but also AzaC treatment resulted in a substantial amount of late apoptotic cells (Fig. 5e). On the proteome level, application of 5.0 µM of either AzaC or AzadC resulted in numerous differentially expressed proteins with DNMT1 being among the top-downregulated proteins after both treatments (Additional file 7: Table S6, Additional file 8: Table S7). As expected, the correlation between the fold changes of the detected proteins after exposure to AzaC or AzadC was very similar (Pearson *r* = 0.5882) and comparable to the corresponding correlation in MOLM-13 (Fig. 4f). However, there was no correlation between the fold changes of the detected proteins when MOLM-13 were compared to HL-60, neither after AzaC (Fig. 5g) nor after AzadC treatment (Fig. 5h), indicating that AzaC and AzadC had very different effects on the MOLM-13 compared to HL-60 on the protein level. IPA confirmed that hypothesis. Neither p53 nor MYC signaling were significantly affected in the HL-60 and importantly not cell death but survival was significantly activated after AzadC treatment (Additional file 9: Table S8). This suggests that in contrast to MOLM-13, HL-60 cells activate genes that promote survival instead of apoptosis when exposed to AzadC, and the higher amount of viable HL-60 even at high AzadC concentrations (Fig. 5d, e) when compared to AzadC-treated MOLM-13 (Fig. 3a) supports this hypothesis.

## Discussion

Our data confirm existing reports that mdC and m^5^C levels are not useful biomarkers whether cancer cells respond efficiently to AzaC or AzadC treatment, especially concerning apoptosis induction. Moreover, we could show that even increased formation of DSBs was insufficient to induce rapid cell death in MOLM-13 after AzaC treatment and in HL-60 after AzaC and AzadC treatment. However, although these markers are not valuable predictors of cell death upon AzaC and AzadC treatment, they can certainly be used to assess whether AzaC and AzadC are taken up by the cells and correctly metabolized, because cellular resistance mechanisms against AzaC or AzadC, e.g., by increased cytidine deaminases or triphosphate hydrolase expression [2], act before the compounds are integrated into DNA and RNA and before DNMT inhibition. The question remains why detrimental effects of DNMT inhibition and thereby formation of severe DNA–protein cross-links can be tolerated in some cases and not in others. Both compounds induce profound and similar cellular changes on the molecular level in MOLM-13. And yet, the phenotypic outcome was completely different with AzaC failing to efficiently induce apoptosis, even at five times higher concentrations compared to AzadC. To understand how the molecular characteristics and the phenotype of cells after AzaC and AzadC treatment are connected, it will be necessary to track AzaC and AzadC metabolism in a holistic approach that uses information from pulse chase experiments and synchronized cells to identify causal time-dependent relationships. In any case it is not sufficient to rely on transcriptome data. Transcriptional downregulation of several key oncogenes with increasing concentration of AzaC resulted in reduced cellular fitness as indicated by the MTT assay but was apparently not sufficient to initiate cell death. Our proteomics data revealed that the translational machinery of MOLM-13 is heavily affected by AzaC but even more by AzadC treatment. Therefore, transcriptome data alone are not enough to estimate the impact of AzaC or AzadC on cancer cells and need to be complemented by proteomics data to gain deeper knowledge about the targeted cellular processes. The only cellular marker in our study that clearly correlated with the failed induction of apoptosis after AzaC treatment in MOLM-13 was the amount of global hmdC. In comparison, AzaC was able to increase hmdC levels and induce apoptosis in HL-60. Compared to MOLM-13, HL-60 were less sensitive to AzadC treatment and many cells resisted apoptosis even at concentrations as high as 5.0 µM, although global hmdC levels were substantially increased. Nevertheless, hmdC was also a valuable biomarker in HL-60 since the minor increase in hmdC as observed after exposure to 1.0 µM of AzadC also correlated with substantially higher cellular fitness compared to exposure to 5.0 µM of AzadC. Moreover, the higher hmdC levels after AzadC treatment also correlated in the HL-60 with higher efficacy to induce apoptosis compared to AzaC. Interestingly, AzaC treatment also restores *TET2* expression and hmdC in a different leukemic context (T-cell acute lymphoblastic leukemia), which results in rapid cell death [41], suggesting that TET activity is an important factor for the efficiency of AzaC or AzadC treatment in inducing cell death. However, whether enhanced TET activity is simply a result of successful cellular reprogramming after AzaC or AzadC treatment or a prerequisite for AzaC- or AzadC-induced cell death of cancer cells, whether there are a specific genomic context and threshold of hmdC formation and why AzaC in contrast to AzadC was not able to restore hmdC in the MOLM-13 remain to be investigated.

## Conclusion

In summary, our study clearly shows that a holistic approach is required to estimate the impact of AzaC and AzadC on cells and to understand the underlying mechanisms that result in a specific phenotypic outcome. It is not sufficient to look at individual aspects like DNA hypomethylation or metabolic activity and to monitor only transcriptome changes or expression changes of single proteins, but not of the whole proteome to predict how well cells respond to AzaC or AzadC treatment. This is especially important to better understand the off-target toxicity of both compounds and to find treatment regimens to avoid them.

## Methods

### Preparation of AzaC and AzadC for cell culture experiments

AzaC (Carbosynth NA02947) and AzadC (Carbosynth NA02969) were purchased and used without further purification. Both compounds were dissolved in Milli-Q H_2_O to a final concentration of 10 mM. Aliquots (10 µL) were made and stored at − 80 °C. Directly before usage, the required volume of 10 mM stock solution was thawed and diluted to 100 µM with Milli-Q H_2_O. The integrity of the compounds was checked on a regular basis using HPLC.

### Cell culture MOLM-13

MOLM-13 cells (Leibniz Institute DSMZ-German Collection of Microorganisms and Cell Cultures; AML cell line) were cultivated at 37 °C in water-saturated, CO_2_-enriched (5%) atmosphere. RPMI 1640 (Sigma-Aldrich R0883), containing 20% (v/v) fetal bovine serum (FBS) (Invitrogen 10500–064) and 1% (v/v) L-alanyl-L-glutamine (Sigma-Aldrich G8541), was used as growing medium. When reaching a density of 2 × 10^6^ cells/mL, the cells were routinely passaged to a density of 0.25–0.4 × 10^6^ cells/mL. Cells were tested at least once in two months for Mycoplasma contamination using Mycoplasma Detection Kit (Jena Bioscience PP-401L).

### Treatment of MOLM-13 with AzaC or AzadC

For all experiments, cells were seeded at a concentration of 0.5 × 10^6^ cells/mL unless stated otherwise and directly treated with AzaC and AzadC using the indicated concentration and incubation time during which the medium was not renewed. Untreated cells served as a negative control in all experiments unless indicated otherwise.

### Isolation of gDNA and UHPLC-QQQ-MS

For the determination of mdC levels, 1.5 × 10^6^ cells were seeded and treated with either 0.5 µM, 1.0 µM or 2.5 µM of AzaC or AzadC for 72 h. After 72 h of incubation time, the cells were harvested, gDNA was isolated and UHPLC-QQQ-MS measurements were performed as previously described in Traube et al. [42] with the modification that 3 µg of gDNA per technical replicate was digested using Nucleoside Digestion Mix (NEB M0649S, 2 µL of nuclease, 1.5 h at 37 °C).

### RNA isolation for RT-qPCR

400 µL of the first flow-through of the gDNA analysis was used to isolate RNA. 300 µL of 95% ethanol was added, transferred to a Zymo-Spin IIC column (ZymoResearch C1011-50) and incubated for 1 min. After RNA binding, the column was centrifuged at RT, 1500 × *g* (2 min) and at 10 000 × g (30 s). The flow-through was discarded, and the column was washed with 800 µL RNA Wash Buffer (ZymoResearch R1003-3). To remove residual DNA contamination, DNA was digested according to the peqGOLD DNase I Digest kit (VWR 13-1091-01), followed by washing steps with 400 µL RNA Prep Buffer (ZymoResearch R1060-2) and 800 µL RNA Wash Buffer. The RNA was eluted using 53 µL of Milli-Q H_2_O, and the concentration was determined using a spectrophotometer.

### cDNA synthesis and RT-qPCR

cDNA synthesis was performed with the iScript cDNA Synthesis Kit (Bio-Rad 1,708,891) according to the manufacturer’s protocol using 1 µg RNA per sample.

For subsequent RT-qPCR, the following oligonucleotides were used (Table 2).

Each primer pair (forward and reverse) was mixed and diluted to a final concentration of 1 µM. Subsequently, per 8 µL of diluted primers, 10 µL of iTaq Universal SYBR Green supermix (Bio-Rad 1,725,124) was added (RT-qPCR reaction mix). RT-qPCR was performed in 20 µL reactions with 2 µL of 10 ng/µL cDNA (20 ng per reaction) mixed with 18 µL of RT-qPCR reaction mix (final primer concentration 400 nm).

All samples were run in technical triplicates using the following PCR program on a qTOWER^3^/G cycler (Jena Biosciences) (Table 3).

**Table 2:** Primers used for RT-qPCR. HK = house keeper

Target	Sequence (5’—3’)
hActB_fw (HK)	GCCGCCAGCTCACCAT
hActB_rev (HK)	CACGATGGAGGGGAAGACG
hBCL-2_fw	ATCGCCCTGTGGATGACTCAGT
hBCL-2_rev	GCCAGGAGAAATCAAACAGAGGC
hMYC_fw	CCTTCTCTCCGTCCTCGGAT
hMYC_rev	CTCATCTTCTTGTTCCTCCTCAGA
hTERT_fw	AAACCTTCCTCAGCTATGCCC
hTERT_rev	GTTTGCGACGCATGTTCCTC
hTET2_fw	AAGGCTGAGGGACGAGAACGA
hTET2_rev	TGAGCCCATCTCCTGCTTCAA

**Table 3:** PCR program used for RT-qPCR

95 °C	03:00 min	
95 °C	00:10 min	40 x
60 °C	00:20 min	
72 °C	00:30 min	
Melt	00:15 min	

An RT-qPCR assay for actin beta (ActB) transcripts was used as a housekeeping transcript reference to calculate ΔCt values. Fold change values were calculated with the ΔΔCt method.

### Isolation of RNA for UHPLC-QQQ-MS

For the determination of m^5^C levels, 6.5 × 10^6^ cells were seeded and treated with either 1.0 µM or 2.5 µM of AzaC or 0.5 µM of AzadC for 72 h. After 72 h of incubation time, the cells were harvested, counted and washed once with Dulbecco’s PBS (Sigma-Aldrich D8537-500ML). Afterward, 1.5 mL of TRI reagent (Sigma-Aldrich T9424-200ML) was added (1 mL for 5–10 Mio cells) to lyse the cells. After 5 min incubation time, 150 µL of 1-bromo-3-chloropropane (Sigma-Aldrich B9673) were added (100 µL per 1 mL TRI reagent) and samples were vortexed thoroughly, followed by an incubation time of 10 min. Afterward, cells were centrifuged at 4 °C, 12,000 × *g* for 15 min. The resulting red phase contained proteins, the interphase the DNA and the upper transparent phase the RNA. For RNA isolation, the upper phase was transferred into a new 1.5 mL tube and 750 µL of 2-propanol (500 µL per 1 mL of TRI reagent) was added. The samples were vortexed thoroughly and incubated for 10 min at RT, followed by centrifugation at 4 °C, 12,000 × *g* for 10 min. The supernatant was discarded, and 1.5 mL 75% (v/v) EtOH (1 mL per 1 mL TRI reagent) was added. After having thoroughly vortexed the sample, a centrifugation step at 4 °C, 12,000 × g for 5 min was performed. The supernatant was carefully discarded, and the pellet was allowed to dry at RT. When the pellet was just about to be completely dry, it was resuspended in 100 µL of Milli-Q H_2_O and the concentration was determined.

tRNA was further purified by size exclusion chromatography (SEC) (AdvanceBio SEC 300 Å, 2.7 μm, 7.8 × 300 mm for tRNA combined with BioSEC 1000 Å) according to our previously published protocol [43]. After purification, the RNA was precipitated and dissolved in 30 μL H_2_O. The RNA concentration of each sample was measured using an Implen nanophotometer.

### RNA UHLPC-QQQ-MS

RNA (100 ng) in aqueous digestion mix (15 µL) was digested to single nucleoside and mixed with metabolically produced stable isotope labeled internal standard as previously described [43]. For quantitative mass spectrometry, an Agilent 1290 Infinity II equipped with a diode-array detector (DAD) combined with an Agilent Technologies G6470A Triple Quadrupole system and electrospray ionization (ESI-MS, Agilent Jetstream) was used.

Operating parameters: positive-ion mode, skimmer voltage of 15 V, cell accelerator voltage of 5 V, N_2_ gas temperature of 230 °C and N_2_ gas flow of 6 L/min, sheath gas (N_2_) temperature of 400 °C with a flow of 12 L/min, capillary voltage of 2500 V, nozzle voltage of 0 V and nebulizer at 40 psi. The instrument was operated in dynamic MRM mode (multiple reaction monitoring, MRM). For separation a Core-Shell Technology column (Synergi, 2.5 μm Fusion-RP, 100 Å, 100 × 2 mm column, Phenomenex, Torrance, CA, USA) at 35 °C and a flow rate of 0.35 mL/min were used in combination with a binary mobile phase of 5 mM NH_4_OAc aqueous buffer A, brought to pH 5.6 with glacial acetic acid (65 μL), and an organic buffer B of pure acetonitrile (Roth, LC-MS grade, purity ≥ 0.99.95). The gradient started at 100% solvent A for 1 min, followed by an increase to 10% over 3 min. From 4 to 7 min, solvent B was increased to 40% and was maintained for 1 min before returning to 100% solvent A and a 3 min re-equilibration period. The sample data were analyzed by MassHunter Quantitative Software from Agilent.

### Isolation of nuclear proteins for western blotting

For the preparation of nuclear extracts, 2.5 × 10^6^ cells were seeded and treated with 0.5 µM, 1.0 µm or 2.5 µM of AzaC or AzadC for 48 h. After treatment, the cells were harvested and nuclear extracts were prepared as previously described by Dignam et al. [44] with the modification that every buffer was supplemented with Phosphatase Inhibitor Cocktail 2 (Sigma-Aldrich P5726) and Phosphatase Inhibitor Cocktail 3 (Sigma-Aldrich P0044), 1:100 each. Afterward, the protein concentration was determined using a Bradford assay (Bio-Rad #5,000,006) as described by the manufacturer. SDS loading buffer (final concentration 50 mM Tris pH 6.8, 100 mM DTT, 2% (w/v) SDS, 10% (v/v) glycerol, 0.1% (w/v) bromophenol blue) was added, and the samples were incubated for 5 min at 92 °C before being stored at − 20 °C. Before loading the samples on a polyacrylamide gel, the samples were heated for additional 2 min at 92 °C and vortexed thoroughly.

### Isolation of whole proteome for western blotting

For the preparation of whole proteome, 2.5 × 10^6^ cells were seeded and treated with 0.5 µM, 1.0 µm or 2.5 µM of AzaC or AzadC for 72 h. After treatment, the cells were harvested and washed once with ice-cold PBS. Afterward, cells were lysed in RIPA buffer (10 mM Tris pH 7.5, 150 mM NaCl, 0.5 mM EDTA, 0.1% SDS, 2 mM MgCl_2_, 0.5 mM DTT, EDTA-free protease inhibitor cocktail tablet (Roche 43,203,100), 1% phosphatase inhibitor cocktail 2 (Sigma P5726-1ML), 1% phosphatase inhibitor cocktail 3 (Sigma P0044-1ML), DNase I, Benzonase) and incubated for 1.5 h on ice. Afterward, the lysate was centrifuged at 4 °C, 12,000 × *g* for 15 min, the supernatant was transferred into a new tube and the protein concentration was determined using a Bradford assay (Bio-Rad #5,000,006) as described by the manufacturer. SDS loading buffer (final concentration 50 mM Tris pH 6.8, 100 mM DTT, 2% (w/v) SDS, 10% (v/v) glycerol, 0.1% (w/v) bromophenol blue) was added, and the samples were incubated for 5 min at 92 °C before being stored at − 20 °C. Before loading the samples on a polyacrylamide gel, the samples were heated for additional 2 min at 92 °C and vortexed thoroughly.

### Western blotting

15 µg of nuclear extract in SDS loading buffer or 30 µg of total protein extract was loaded on a 4–15% precast polyacrylamide gel (Bio-Rad #4561083EDU), and Color-coded Prestained Protein Marker, Broad Range (10–250 kDa) (New England Biolabs P7719S or Bio-Rad 1,610,374) was used as a protein standard. The gel was run at constant 150 V for 60 min in SDS running buffer (25 mM Tris, 192 mM glycine, 0.1% (w/v) SDS). For blotting, we used a PVDF blotting membrane (GE Healthcare Amersham Hybond P0.45 PVDG membrane 10,600,023) and pre-cooled Towbin blotting buffer (25 mM Tris, 192 mM glycine, 20% (v/v) methanol, 0.038% (w/v) SDS). The membrane was activated for 1 min in methanol, washed with Milli-Q water and equilibrated for additional 1 min in Towbin blotting buffer; the Whatman gel blotting papers (Sigma-Aldrich WHA 10,426,981) were equilibrated for 15 min in Towbin buffer and the precast gel was equilibrated for 5 min in Towbin buffer after the run. Western blotting (tank (wet) electro transfer) was performed at 4 °C for 9 h at constant 35 V or for 4 h at constant 50 V. After blotting, the PVDF membrane was blocked for 0.5—1 h at room temperature using 5% (w/v) milk powder in TBS-T (20 mM Tris pH = 7.4, 150 mM NaCl, 0.1% (v/v) Tween-20). The primary antibodies were diluted in 5 mL of 5% (w/v) milk powder in TBS-T. The blocking suspension was discarded, and the diluted primary antibodies were added for 12 h at 4 °C and shaking. After incubation, the primary antibodies were discarded, and the membrane was washed three times ten minutes with TBS-T. HRP-conjugated secondary antibodies were diluted in 5% (w/v) milk powder in TBS-T and added for 1 h at room temperature under shaking. Afterward, the membrane was washed two times with TBS-T and one time with TBS (TBS-T without Tween-20) before SuperSignal West Pico Chemiluminescent Substrate (Thermo Scientific 34,077) was used for imaging. Western blots were imaged using Amersham Imager 680 (auto exposure mode).

For imaging the same blot multiple times using different antibodies, the membrane was directly stripped after imaging. To this end, the membrane was put in TBS-T and the buffer was heated in a microwave until boiling. Afterward, the buffer was discarded and the procedure was repeated in total three times. After stripping, the membrane was blocked again using 5% (w/v) milk powder in TBS-T and the protocol followed the above-described procedure.

*Primary antibodies*

- Anti-phospho-Histone-H2AX (γH2AX, Ser-139) antibody, Millipore 05-636-1 clone 7BW301, mouse monoclonal antibody, 1:1000
- Anti-Histone-H3 antibody, Cell Signaling Technology 4499S clone D1H2, rabbit monoclonal antibody, 1:1000
- Anti-c-MYC antibody, ptglab 67,447-1-Ig, mouse monoclonal antibody, 1:500
- Anti-HOXA9 antibody, ptglab 18,501-1-AP, rabbit polyclonal antibody, 1:1000
- Anti-BCL-2 antibody, ptglab 12,789-1-AP Lot 00,087,156, rabbit polyclonal antibody, 1:1000
- Anti-phospho-TP53 (Ser15) antibody, ptglab 28,961-1-AP, rabbit polyclonal antibody, 1:1000

*Secondary antibodies*

- HRP-conjugated anti-mouse IgG, Sigma-Aldrich AP130P, 1:5000
- HRP-conjugated anti-rabbit IgG, Sigma-Aldrich A0545, 1:5000

### Brightfield microscopy

For the brightfield microscopy images, cells were imaged directly in the medium after 72 h treatment with 0.5 µM, 1.0 µM or 2.5 µM of either AzaC or AzadC using an EVOS FL microscope (ThermoFisher) in the transmission mode and 40 × magnification.

### MTT assay

For the MTT assay, 5 × 10^4^ cells were seeded in 100 µL RPMI/20% FBS medium that did not contain phenol red. The assay was performed as described previously [45]. Each time point (24 h, 48 h or 72 h) included samples for untreated cells and cells that were treated with 0.5 µM, 1.0 µM or 2.5 µM of either AzaC or AzadC. Each time point was measured individually and after measuring the absorption at 570 nm, the average of the technical replicates was calculated and the absorption of the AzaC- or AzadC-treated cells was set in relation to the absorption measured for the untreated cells, resulting in the relative metabolic activity. Three biologically independent experiments were performed per time point, and each sample was measured in technical quadruplicates.

### Flow cytometry analysis for monitoring proliferation

To measure proliferation, EdU Flow Cytometry 488 kit (baseclick BCK-FC488-50) was used according to the manufacturer’s protocol. In brief, MOLM-13 (1 × 10^6^ per condition, 0.5 × 10^6^/mL) were treated with 0.5 µM, 1.0 µM or 2.5 µM of either AzaC or AzadC for 48 h, before EdU (final concentration 10 µM) was added for two additional hours. Untreated cells without EdU served as unstained control, and untreated cells with EdU served as untreated control. Afterward, the cells were harvested and washed with DPBS, including 1% (w/v) BSA, before fixation and permeabilization. Then, click chemistry was performed as described in the manual. Prior to flow cytometry measurement, the cells were filtered through a 35-µm strainer. For the analysis, BD FACSCanto™ and FlowJo Single Cell Analysis Software (v10.8.0) were used. Gates were set once for the control sample and then applied to all other samples.

### Flow cytometry analysis for monitoring cell death

Previous to flow cytometry analysis, cells were treated with 0.5 µM, 1.0 µM or 2.5 µM of either AzaC or AzadC for 72 h, harvested and washed twice with DPBS. Untreated cells served as a control. Afterward, cells were counted and 2 × 10^5^ cells per sample were transferred into a new tube. Apoptosis and necrosis were determined by using the FITC Annexin V Apoptosis Detection Kit (BioLegend 640,914) and SYTOX™ Red Dead Cell Stain (ThermoFisher S34859). To this end, cells were resuspended in 100 µL of Annexin V binding buffer supplemented with 1 µL of FITC-conjugated Annexin V, gently vortexed and incubated at RT for 15 min in the dark. Afterward, cells were put on ice and 100 µL of cell suspension (2 × 10^5^ cells) were filtered through a 35 µm strainer. 0.2 µL of Sytox was added just before the measurement. For the analysis, BD FACSCanto™ and FlowJo Single Cell Analysis Software (v10.8.0) were used. Gates were set once for the control sample and then applied to all other samples.

### Proteomics

MOLM-13 cells were incubated for 72 h with 0.5 μM AzadC, 1 μM or 2.5 μM AzaC in 4 replicates each. Untreated cells (*n* = 4) served as a control. The cells were harvested and washed twice with PBS. Sample preparation followed in principle a previously published protocol using filter-assisted sample preparation (FASP) [46]. Lysis was achieved by adding 1 mL 100 mM Tris/HCl pH 8.5, 8 M urea, incubation at 95 °C for 5 min and subsequent sonication at 20% intensity for 20 s using a rod sonicator. The suspension was centrifuged at 14,000 × *g* for 5 min, and the resulting supernatant was transferred into a new reaction tube. A BCA assay was conducted to determine the protein concentration, before a defined volume (e.g., 700 μL) was taken from each sample and mixed with TCEP (10 mM final concentration) and 2-chloroacetamide (40 mM final concentration) before incubation at 95 °C for 5 min. 30 μg of each sample was filled up to 150 μL with 100 mM Tris/HCl pH 8.5, 8 M urea and transferred onto a 30 kDa molecular weight cutoff column (Microcon-30, *Merck Millipore*). 150 μL 100 mM Tris/HCl pH 8.5, 8 M urea was added to the column before centrifugation at 14,000 × *g* for 15 min. The flow-through was discarded, and the columns were washed twice by addition of 100 μL 50 mM ammonium bicarbonate and subsequent centrifugation at 14,000 × *g* for 10 min. The filter units were then placed into a new collection tube before addition of 100 μL 50 mM ammonium bicarbonate containing trypsin (1:100 trypsin/protein) and incubation at 37 °C overnight. Next, the samples were centrifuged at 14,000 × *g* for 15 min followed by washing the column twice with 40 μL 50 mM ammonium bicarbonate and centrifugation at 14,000 × *g* for 10 min. The eluate was acidified with 5% formic acid to a pH of 1–2 and purified using C_18_ cartridges (Sep-Pak tC18 1 cc, 50 mg, *Waters*) and a vacuum manifold. For this, the cartridges were washed with 1 mL MeCN, 1 mL 80% MeCN, 0.5% formic acid and thrice with 1 mL 0.5% formic acid, applying vacuum after each step. Then, the acidified samples were loaded onto the cartridges without application of vacuum following three washing steps using 1 mL 0.5% formic acid. Clean collection tubes were inserted into the vacuum manifold, and the peptides were eluted by addition of 250 μL 80% MeCN, 0.5% formic acid without applying vacuum before another 250 μL of this buffer was added while applying vacuum. Finally, the solvent was evaporated and the purified peptides were resuspended in 2% MeCN, 0.1% formic acid.

2 μg of each sample was submitted to a LC-MS analysis using an *Ultimate 3000 RSLCnano* UHPLC (*Thermo Fisher Scientific*) coupled to an *Orbitrap Eclipse* mass spectrometer (*Thermo Fisher Scientific*) with the *FAIMS Pro* interface attached. First, the peptides were separated by reverse-phase chromatography using a pre-concentration setup. For this, the samples were bound to a pre-column (*Acclaim C18 PepMap100*, 300 μm i.d., 5 mm length; *Thermo Fisher Scientific*) and then eluted onto the analytical column (*SilicaTip Emitter*, 75 µm i.d., 8 µm tip, 15 cm length; *New Objective*; packed in-house with *ReproSil-Pur 120 C18-AQ, *1.9 µm, 120 Å; *Dr. Maisch GmbH*), which was heated to 40 °C using a column oven by *Sonation*. The separation was achieved at a flow rate of 0.3 μL/min and application of a gradient between solvent A (0.1% formic acid in H_2_O) and solvent B (0.1% formic acid in MeCN) going from 7 to 24.8% B in 99 min and from 24.8 to 35.2% B in 21 min. The ionization was carried out by applying a voltage of 2.0 kV to the column.

The eluting peptides were analyzed at alternating FAIMS CV voltages of − 50 V and − 70 V in *Standard Resolution* mode with the total carrier gas flow set to static and 3.5 L/min. To take peptide charge-state distributions into account, the data-dependent acquisition at − 50 V was run for 1.7 s, while the corresponding cycle time at − 70 V was shortened to 1.3 s. The rest of the parameters were kept identical for the two CV values, starting with a full mass orbitrap scan in profile mode at a resolution of 240,000 using a mass range of 375–1500 m*/z* and a RF lens level of 30%. The AGC target was set to *Standard*, and the maximum injection time was set to 50 ms. Following this scan, multiple data-dependent MS2 scans were carried out for the cycle time specified above using the filters *MIPS* (*Peptide*), *Intensity* (intensity threshold: 1.0e4), *Charge State* (include charge states 2–6, don’t include undetermined charge states) and *Dynamic Exclusion* (exclude for 40 s after one selection with a window of ± 10 ppm, also exclude isotopes and other charge states). The most intense ions were individually selected using an isolation window of 1,2 m*/z*, subjected to fragmentation at a normalized HCD energy of 30% and analyzed in the ion trap at *Rapid* scan rate with a *Normal* mass range, the AGC target set to *Standard* and a maximum injection time of 50 ms.

Identification and label-free quantification (LFQ) of peptides and proteins was accomplished using the MaxQuant software version 2.0.3.0 [47, 48]. To prepare the .RAW-files for analysis, they were split into two files containing either the spectra obtained at a CV of − 50 V or − 70 V. This was accomplished by the *FAIMS MzXML Generator* software developed by the *Coon lab* [49]. During the MaxQuant analysis, these two files were handled like fractions. Next, a database containing the human proteome was chosen. As protease, Trypsin was chosen and a maximum of two missed cleavages were allowed. Carbamidomethylation of C was set as static modification, while oxidation of M and phosphorylation of YST were set as dynamic modification. Peptide mass deviations were set to 20 ppm in the first search and 4.5 ppm in the second search, respectively. The minimal peptide length was set to 6, and the PSM and protein FDRs were both set to 0.01. A reverse of the database was chosen as decoy database. The LFQ option was checked and the minimum ratio count was set to 2. The feature “match between runs” was checked, and a match time window of 0.7 min as well as an alignment time window of 20 min was defined. MaxQuant results were analyzed using Perseus (v 1.6.14) [50]. In short, contaminants that were assigned by MaxQuant were filtered and LFQ intensities were log_2_ transformed (Perseus step1). The four biological replicates (cells that were treated the same way) were grouped, and afterward, the data were filtered so that only proteins remained in the data set which were detected in at least three biological replicates of at least one group to ensure high quality data (Perseus step2). Afterward, the remaining missing values were imputed using imputation from Gaussian distribution (per column) with the default settings (Perseus step3). Last, the volcano plot option was used to display pairwise comparisons (Perseus step4).

### IPA

Proteomics data were further analyzed with the use of QIAGEN IPA (QIAGEN Inc., https://digitalinsights.qiagen.com/IPA). For IPA [31], processing of the proteomics data was processed until the end of Perseus step 2. Before imputation, however, proteins were checked groupwise and if a protein was not detectable in any of the four biological replicates of one group, indicating that the protein was not present in this group, an artificial log_2_ transformed LFQ intensity of 1 was assigned. Afterward, the remaining missing values were imputed with the values that were obtained from the initial imputation of Perseus step 3. Last, treated samples were pairwise compared to the untreated control using the volcano plot option and the obtained p values and log2 fold changes were used for IPA. In IPA, core analysis was performed for each comparison with the setting “human” as species and a *p* value cutoff of 0.05.

### Statistical analysis

Statistical analysis, except for the proteomics data where Perseus was used, was performed using GraphPad Prism (v 9). Per biological replicate, the mean of the technical replicates was calculated where applicable and only the biological replicates were taken into account for the statistical analysis.

### HL-60 cells

Maintenance and treatment of HL-60 (ATCC) as well as the described experiments were equally performed as with MOLM-13.

## Supplementary Information

**Additional file 1**. Supplementary information.

**Additional file 2.** Proteomics data MOLM-13 1.0 μM AzaC.

**Additional file 3.** Proteomics data MOLM-13 2.5 μM AzaC.

**Additional file 4.** Proteomics data MOLM-13 0.5 μM AzadC.

**Additional file 5.** Proteomics data MOLM-13 comparison AzaC and AzadC.

**Additional file 6.** Proteomics data MOLM-13 IPA.

**Additional file 7.** Proteomics data HL-60 5.0 μM AzaC.

**Additional file 8.** Proteomics data HL-60 5.0 μM AzadC.

**Additional file 9.** Proteomics data HL-60 IPA.

**Additional file 10.** Statistical analysis mdC data MOLM-13.

**Additional file 11.** Statistical analysis m^5^C data MOLM-13.

**Additional file 12.** Statistical analysis MTT assay MOLM-13.

**Additional file 13.** Statistical analysis flow cytometry cell death data MOLM-13.

**Additional file 14.** Statistical analysis flow cytometry apoptotic cells MOLM-13.

**Additional file 15.** Statistical analysis mdC data HL-60.

**Additional file 16.** Statistical analysis hmdC data HL-60.

## Data Availability

The mass spectrometry proteomics data of the MOLM-13 have been deposited to the ProteomeXchange Consortium via the PRIDE [51, 52] partner repository with the dataset identifier PXD031455. The UHPLC-QQQ-MS mdC and hmdC data, the RT-qPCR data, the flow cytometry data and the HL-60 proteomics data have been deposited in the LMU Open Data repository with the identifier https://doi.org/10.5282/ubm/data.314.
