# Supplementary material for: Comprehensive comparison between azacytidine and decitabine treatment in an acute myeloid leukemia cell line
Source: Clin Epigenetics. 2022 Sep 11;14:113. doi: 10.1186/s13148-022-01329-0 (PMC9465881; doi:10.1186/s13148-022-01329-0)
Supplement: Supplementary file 1 — Additional file 1. Supplementary information. [file 13148_2022_1329_MOESM1_ESM.pdf]

Supporting Information

Comprehensive comparison between azacytidine and decitabine treatment in an acute myeloid leukemia cell line

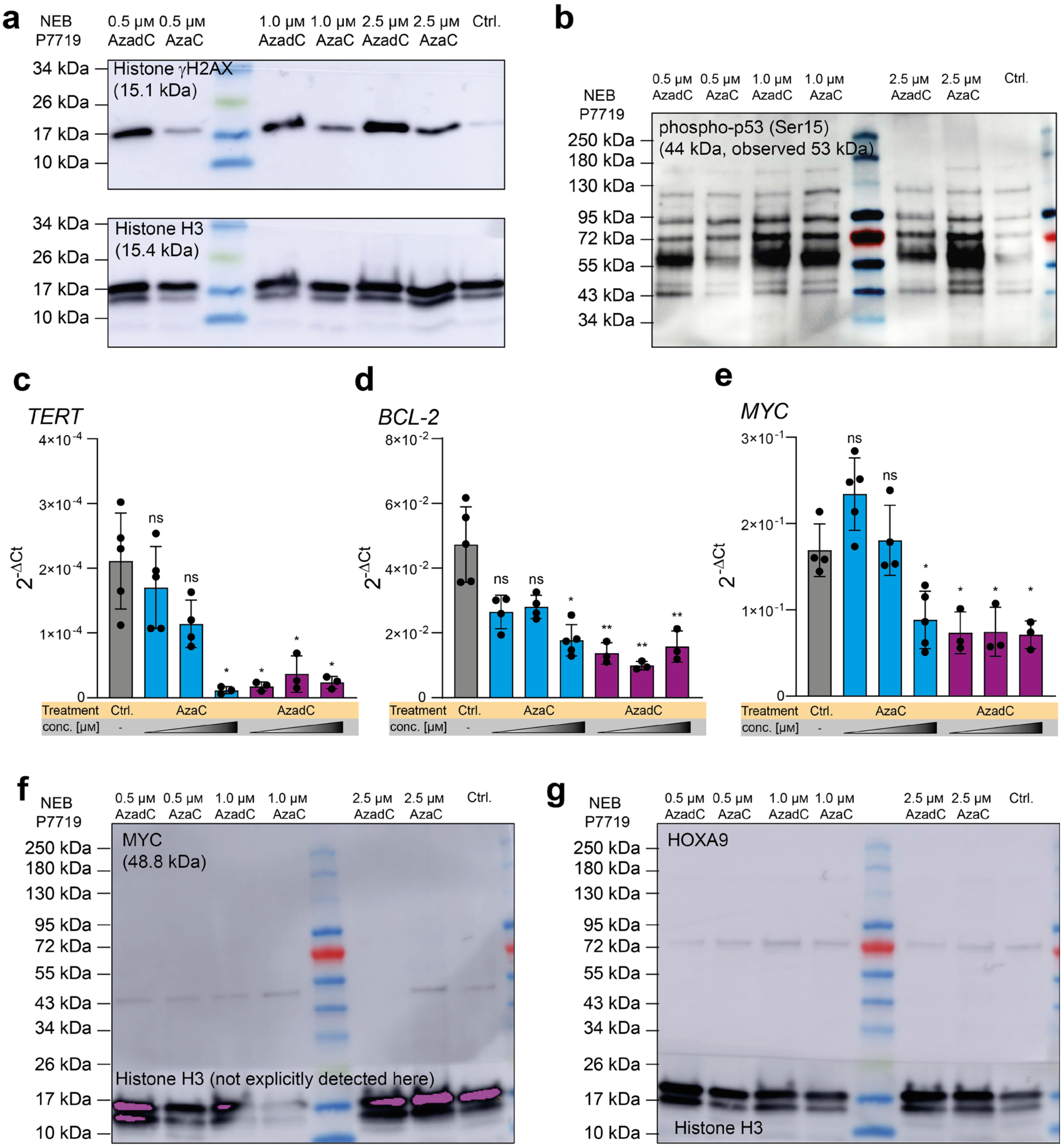

Figure S1: **Detailed immunoblots and ΔC_t of the RT-qPCRs. a** Detailed immunoblot of 48 h treated MOLM-13 (nuclear lysate) of Fig. 1b (upper part), including the protein ladder. First, γH2AX was detected. Afterwards, the blot was stripped and histone H3 was detected as a loading ctrl. **b** Detailed immunoblot of 48 h treated MOLM-13 (nuclear lysate) of Fig. 1b (lower part), including the protein ladder. The blot was stripped after HOXA9/H3 and MYC detection (Fig. S1f,g) and then phospho-p53 (Ser15) was detected. The histone H3 loading ctrl. is the one of Fig. S1f and was therefore not imaged again. **c – e** $2^{-\Delta Ct}$ results of the RT-qPCRs in Fig. 1d – f. The triangle indicates increasing concentrations of AzaC or AzadC (0.5 µM, 1.0 µM and 2.5 µM). Transcripts of interest (TOI) is *TERT* for **c**, *BCL-2* for **d**, and *MYC* for **e**, house-keeping gene (HK) is Actin β**.** ΔCt = TOI – HK. Statistical analysis Ordinary one-way ANOVA combined with Dunnett's multiple comparisons test. ns $p_{adj} \geq 0.05$; * $p_{adj} < 0.05$, ** $p_{adj} < 0.01$. **f, g** Detailed immunoblot of 48 h treated MOLM-13 (nuclear lysate) of Fig. 1g, including the protein ladder. HOXA9 and the loading Ctrl. H3 were detected in parallel (Fig. S1f), then the upper part of the blot was stripped and MYC was detected (Fig. S1g).

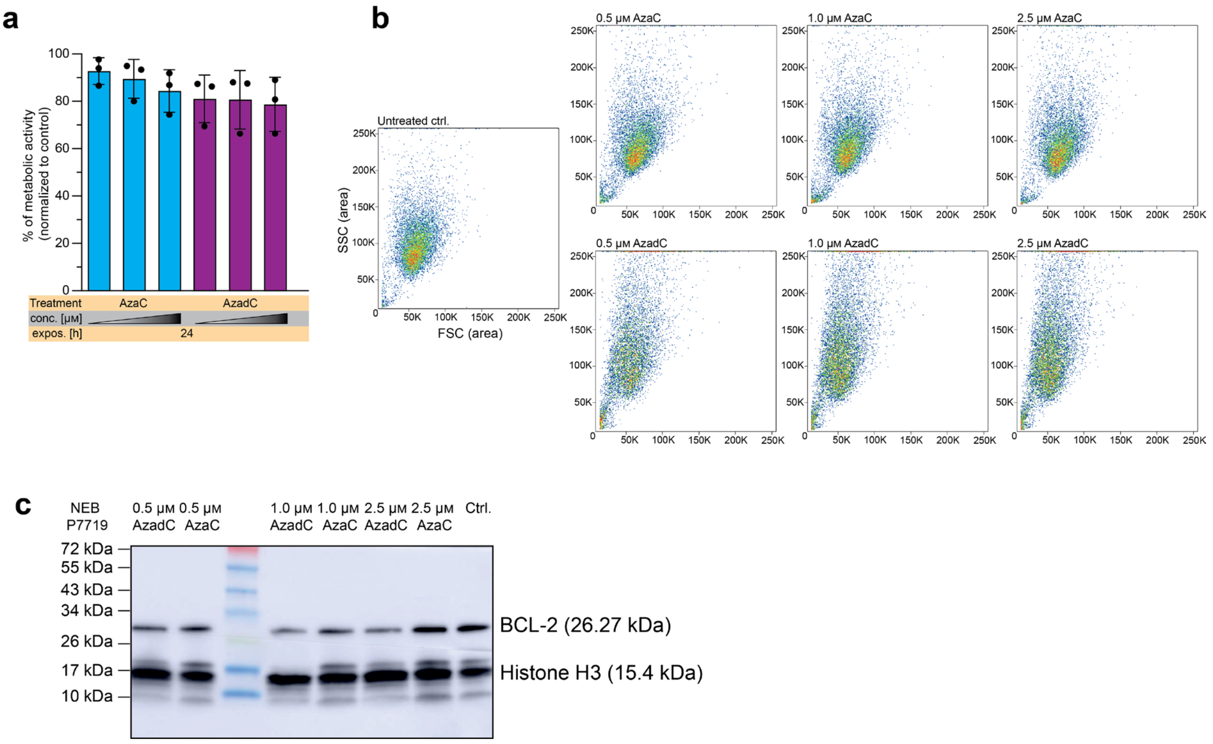

Figure S2: **Cell death analysis of AzaC and AzadC-treated MOLM-13**. **a** MTT assay results of MOLM-13 relative to the untreated control after 24 h The triangle indicates increasing concentrations of AzaC or AzadC (0.5 µM, 1.0 µM and 2.5 µM). Flow cytometry analysis of MOLM-13 after increasing concentrations of AzaC or AzadC. Depicted is FSC-A vs SSC-A. **c** Detailed immunoblot 72 h treated MOLM-13 (whole lysate) of Fig. 3d, including the protein ladder. BCL-2 and the loading ctrl. histone H3 were detected in parallel.

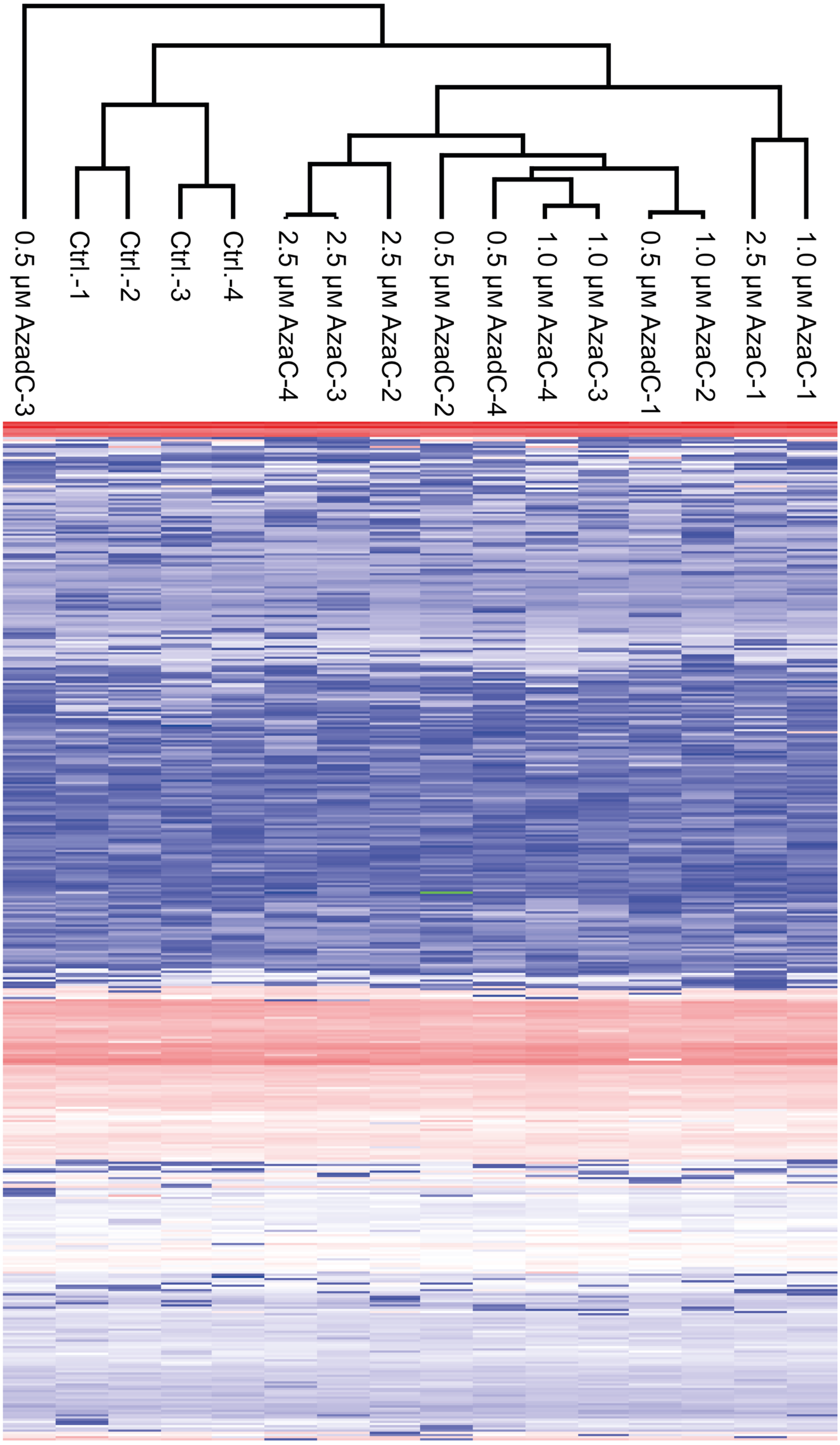

Figure S3: **Hierarchical clustering of MOLM-13 proteomics data sets.** The heat map was calculated in Perseus using Euclidian distance.

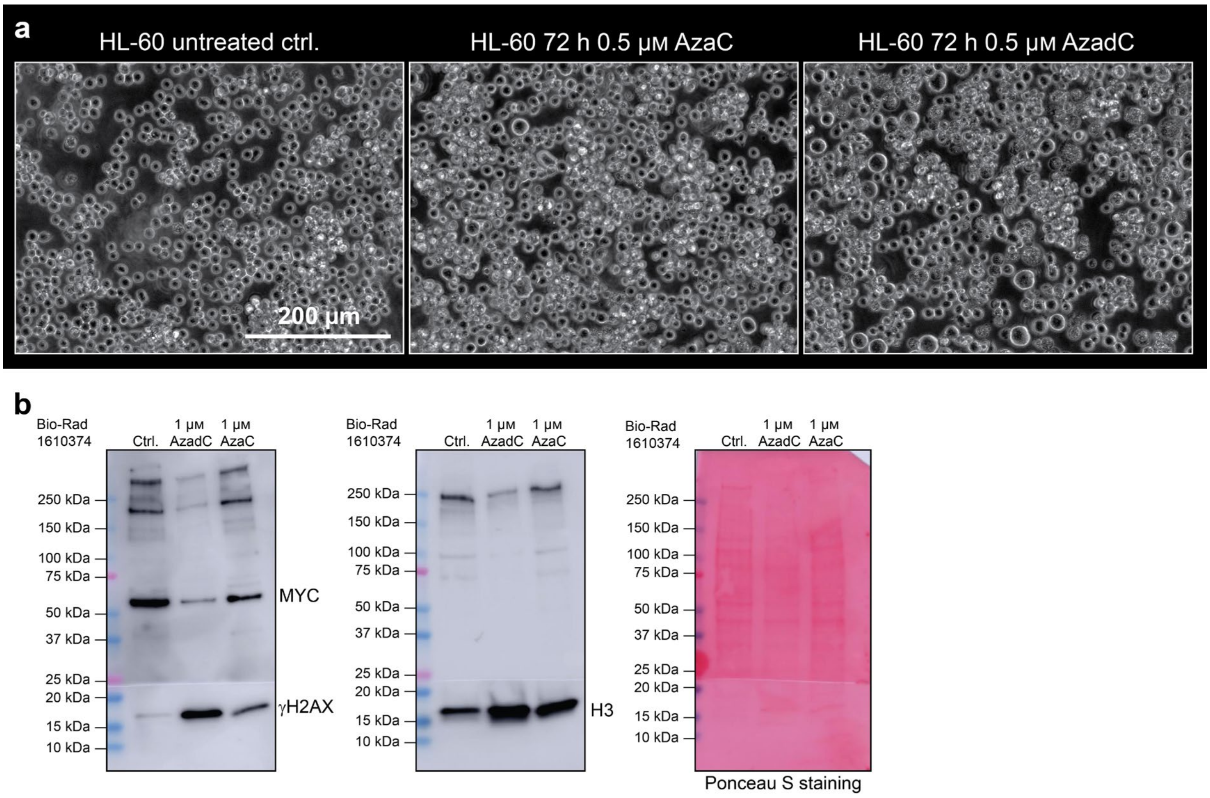

Figure S4: **Additional data sets for AzaC or AzadC treated HL-60**. **a** Brightfield microscopy images of HL-60 that were treated for 72 h with either 0.5 µM of AzaC or 0.5 µM of AzadC. Untreated cells served as a control. **b** Detailed immunoblot of 72 h treated HL-60 (nuclear lysate) of Fig. 5c, including the protein ladder. First, γH2AX and MYC were detected. Afterwards, the blot was stripped and histone H3 was detected as a loading ctrl. Ponceau S staining served as an additional loading control.
